# Protein-based nanocarrier delivering kartogenin derivative to cartilage matrix for intra-articular treatment of osteoarthritis

**DOI:** 10.1016/j.jddst.2026.108162

**Published:** 2026-02-28

**Authors:** Luca Morici, Sébastien Jenni, Bill Hakim, Carlos Rodríguez-Nogales, Eric Allémann, Ambika G. Bajpayee, Olivier Jordan

**Affiliations:** a School of Pharmaceutical Sciences, University of Geneva, Rue Michel-Servet 1, Geneva, 1211, Switzerland; b Institute of Pharmaceutical Sciences of Western Switzerland, Rue Michel-Servet 1, Geneva, 1211, Switzerland; c Department of Bioengineering, Northeastern University, 805 Columbus Avenue, Boston, MA, 02120, USA

**Keywords:** Avidin, Osteoarthritis, Cartilage, Kartogenin, Protein-based delivery system, Nanocarrier

## Abstract

Osteoarthritis is the most common degenerative joint disease worldwide, yet no intra-articular disease-modifying osteoarthritis drugs (DMOADs) have succeeded in clinical trials due to poor therapeutic outcomes and limited cartilage targeting. Positively charged nanosized carriers have been shown to penetrate the dense, negatively charged cartilage. Here, we synthesized a cationic avidin-biotin-PEG_2_-kartogenin (Av-bKGN) nanocarrier in a rapid, scalable manner. Over 80 % of Av-bKGN remained stable after 24 h in PBS and simulated synovial fluid, whereas a new kartogenin derivative was predominantly generated in esterase-rich media. Av-bKGN suppressed inflammatory mediators (NO^−^_2_ , IL-6) in human chondrocytes and macrophages, and its nanosize (8.7 ± 0.26 nm) and positive charge (+18.3 ± 0.75 mV) enhanced cartilage uptake by 4.3- and 11.4-fold, respectively, and cartilage retention by 2-fold over 10 days in PBS and simulated synovial fluid compared to its neutral counterpart, neutravidin. A custom diffusion chamber showed Av-bKGN diffuses tenfold slower than neutravidin, driven by electrostatic binding to cartilage glycosaminoglycans (GAGs). Combining avidin’s delivery platform with bKGN’s DMOAD activity reduced GAG loss in *in vitro* IL-1a-stimulated OA model. A single Av-bKGN dose provided protection equivalent to multiple free-drug doses (25 % reduced GAG loss), with repeated Av-bKGN doses achieved 1.7-fold greater chondroprotection. These findings highlight electrostatic interaction-driven uptake and retention of Av-bKGN as a promising strategy for targeted cartilage therapy.

## 1. Introduction

Osteoarthritis (OA) is the most common degenerative joint disease worldwide [1]. The hip and the knee are the most affected joints, having the heaviest burden on the healthcare costs. Cartilage degradation is one of the most critical aspects of OA physiopathology associated with joint inflammation and subchondral bone remodeling. This results in chronic pain in patient with an impairment in walking and a decreased quality of life. At present, only symptomatic treatments are available to relieve pain and decrease joint inflammation. No disease-modifying osteoarthritis drugs (DMOADs) have passed clinical trials conducted under the US Food and Drug Administration (FDA) or the European Medicines Agency (EMA) because of the low therapeutic outcomes, systemic toxicity and lack of cartilage targeting [2,3]. DMOADs, defined as drugs able to stop or revert the process of OA and restore the functionality of the articular joint, mostly act on chondrocyte pathways as pro-anabolic or anti-catabolic drugs [4,5].

Kartogenin (KGN), a pro-anabolic compound discovered in 2012 [6], showed promising chondrogenic properties by stimulating chondrocyte differentiation from human mesenchymal stem cells (hMSCs) at low concentrations (median effective concentration of 100 nM). The chondroprotective effect of KGN on chondrocytes was also proved in OA *in vivo* models. The mechanism of action of KGN elucidated by Johnson et *al.* [6] involves the intracellular RUNX 1 pathway of chondrocytes. First, the KGN is taken up by chondrocytes to bind the filamin A which normally interacts with the core-binding factor β subunit (CBFβ). Then the CBFβ subunit translocates to the cellular nucleus and interacts with the RUNX 1. Finally, the CBFβ-RUNX1 complex upregulates some transcriptional genes involved in the production of aggrecans and type II collagen, which are the principal components of cartilage extracellular matrix (ECM) [7].

The administration of KGN via the intra-articular (IA) route is limited by its low water solubility, and short retention time in the joint. Formulating KGN nanocrystals improves the water solubility of drugs and enables larger doses to be delivered. When combined with drug delivery systems, such as polymeric macroparticles or hyaluronic acid, the joint retention time is prolonged [8–10]. Additional delivery systems that respond to physiological cues, such as thermal-responsive PNI-MAP-based microgels [11,12] and pH-responsive nanocarriers [13], are emerging for joint lubrication and drug release for OA treatment.

Nevertheless, the excessively large size of these delivery systems is responsible for the lack of cartilage penetration [14–16]. Since the chondrocytes are mostly located in the deep zone of the ECM, nanosized delivery systems are required to penetrate the full thickness of cartilage [17,18]. Only delivery systems having a nanoscale size below 10 nm were reported to be able to penetrate through healthy cartilage pores [19]. Over OA progression, the cartilage matrix permeability increases, with pore size reaching 100 nm. Avidin, a cationic protein, was reported to be a potential cartilage-penetrating nanocarrier which may interact electrostatically with the anionic glycosaminoglycans (GAGs) [20,21]. A previous study by He et *al.* [22] explored the use of avidin as a cationic, multi-arm nanocarrier for delivering KGN, showing promising *ex-vivo* results. The cartilage-targeting capability of multi-arm avidin was further confirmed *in vivo*, showing full-thickness cartilage penetration and retention for up to 7 days following intra-articular injection [23].

Another challenge in translating KGN as a DMOAD for intra-articular delivery lies in its stability within the synovial fluid (SF). During OA progression, inflammatory processes alter the joint milieu, potentially compromising drug bioactivity. The enzymatic activity of esterases in the synovial joint during OA can affect the pharmacokinetics of free DMOADs, as well as those chemically bound to delivery systems/nanocarriers, thereby influencing their therapeutic output [24,25]. Therefore, this study evaluated KGN stability under simulated SF conditions and in esterase-rich media to mimic the arthritic environment of the joint.

The main objectives of the present study were: i) to define a two-step rapid and scalable synthesis of biotin-PEG_2_-KGN and to formulate and characterize the physicochemical properties of the tetrameric avidin- biotin-PEG_2_-KGN conjugate; ii) to perform a metabolization study in simulated SF conditions and in esterase-rich media; iii) to evaluate the *in vitro* anti-inflammatory properties on human chondrocytes and murine macrophages; iv) to measure intra-cartilage uptake and retention in physiological saline and simulated SF environments; v) to measure real-time one-dimensional diffusion/transport via a transport chamber [26] and vi) to quantify *ex vivo* the chondroprotective properties (GAGs loss) of the tetrameric avidin-biotin-PEG_2_-KGN conjugate.

## 2. Experimental section

### 2.1. Materials

Phthalic anhydride, N,N-Diisopropylethylamine (DIPEA) were purchased from Acros Organics (Geel, Belgium) and Biotin-PEG_2_-OH from PurePEG LCC (San Diego, CA, USA). 4-aminobiphenyl, N,N-Dimethylformamide (99.8 %), Dimethyl sulfoxide (99.9 %), acetonitrile for HPLC (99.9 %), (benzotriazol-1-yl-oxy)tripyrrolidinophosphonium hexafluorophosphate (PyBOP), esterase from porcine liver (≥15 units/mg), human serum albumin (HAS), Biotin (5-fluorescein), 1,9-dimethyl-methylene blue zinc chloride double salt, sodium chloride, glycine, sodium azide, hydrochloric acid solution and non-fat-dried milk were supplied from Sigma Aldrich (Sant Louis, MO, USA). Phosphate buffer saline (PBS) solution, Pierce^™^Biotin Quantification Kit, 4-Dimethylaminopyridine (DMAP), NeutrAvidin^™^ Biotin binding protein and Protease Inhibitor Mini Tablets were acquired from Thermo Fisher Scientific (Rockford, IL, USA). Trifluoroacetic acid (99 %) was purchased from Alfa Aesar (Kandel, Germany) while formic acid was acquired from Reactolab SA (Servion, Switzerland). Avidin was obtained from Merck KGaA (Darmstadt, Germany). The simulated synovial fluid was ordered from Biochemazone (Alberta, Canada). The simulated SF was chosen due to its similarity composition to real SF. The supplier (Biochemazone) reported the following composition: salts (PO_4_^3-^ , Na^+^, K^+^, Ca^2+^, Cl^−^ ), amino acids, lipids and phospholipids such as dipalmitoylphosphatidylcholine (DPPC), serum proteins (such as γ-globulins and albumin) (5 mg/mL), glucose, vitamins, mucoprotein, sialic acid, hyaluronic acid (1-3 mg/mL), urea and lubricants, as well as viscosity enhancers and a buffered solution making it isotonic with a pH of 7.4 and a viscosity of 0.1–0.5 P s. Simulated SF contains no immunological components.

The fresh bovine cartilage was obtained from a local butcher shop Research 87 (Boylson, MA, US). 1400W, an inducible nitric oxide synthase inhibitor (iNOS) was ordered from Abcam (Cambridge, UK). Human recombinant IL-1α was obtained from Peprotech (Rocky Hill, NJ, USA) while High glucose Dulbecco’s Modification of Eagle’s Medium (DMEM) was obtained from Cellgro (Manassas, VA, USA). HEPES, non-essential amino acids (NEAA), penicillin-streptomycin Antibiotic-Antimycotic (PSA), trypsin-EDTA and Insulin-Transferrin-Selenium (ITS) were purchased from Gibco (Carlsbad, CA, USA). Ascorbic acid and L-proline were from Fisher Bioreagents (Pittsburgh, PA, USA).

### 2.2. Synthesis of biotin-PEG_2_-kartogenin (bKGN)

**Fig. 1.**
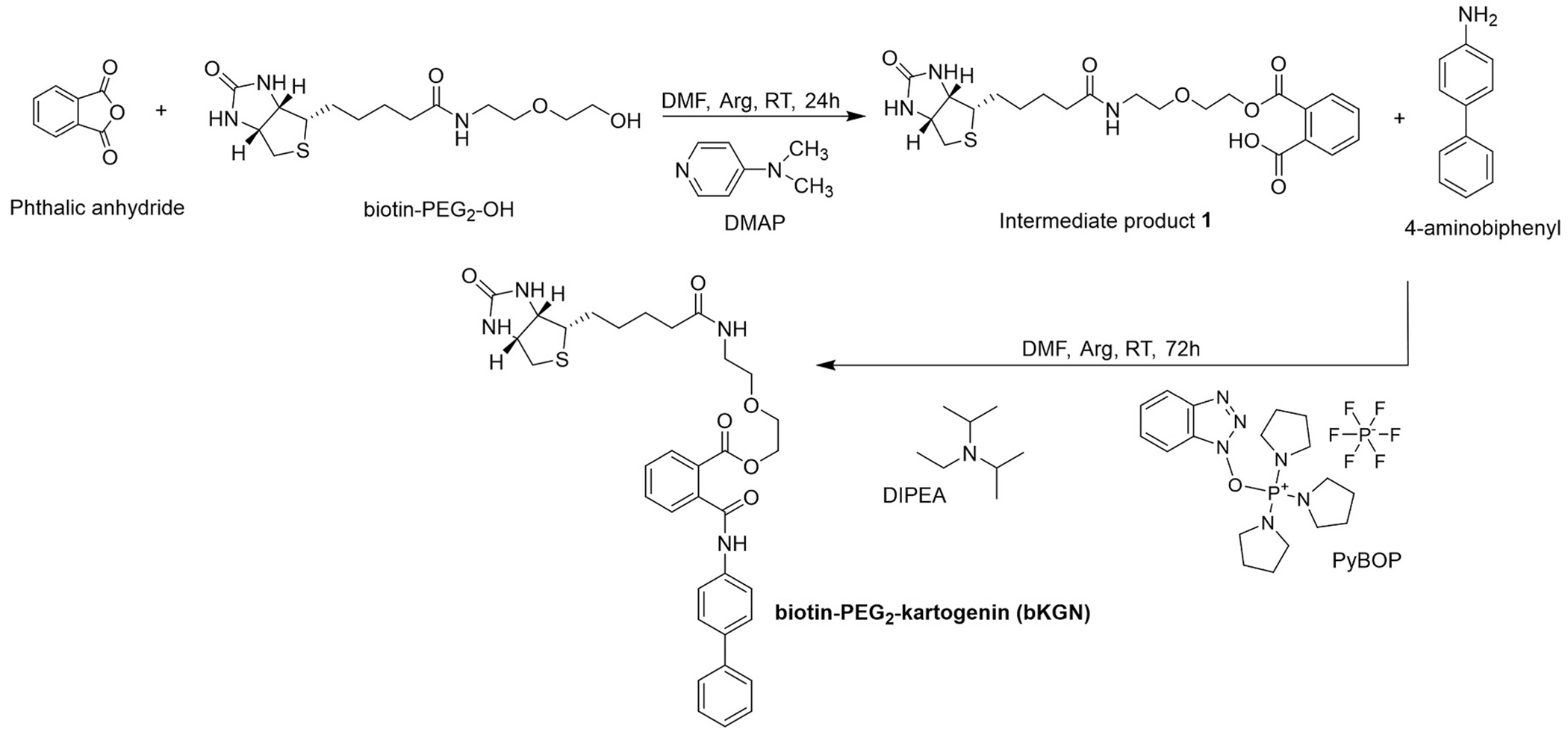
Schematic representation of the 2-step synthesis of bKGN.

The biotin-PEG_2_-OH (50 mg,1 Eq.) was stirred for 24 h with phthalic anhydride (2 Eq.) in 5 mL of DMF in the presence of 4-(dimethylamino) pyridine (DMAP) (0.5 Eq.) under Argon (Ar) atmosphere at RT to obtain the intermediate product **1** (68.2 mg, yield of 88 %)(Fig. 1).

Then bKGN was synthesized via the formation of an amide bond in 5 mL of DMF between the intermediate product (1 Eq.) and the 4-aminobiphenyl (1.25 Eq.) using benzotriazol-1-yloxytripyrrolidinophosphonium hexafluorophosphate (PyBOP) (1.25 Eq.) as coupling reagent and N, N-diisopropylethylamine (DIPEA) (3 Eq.) as base. The reaction was stirred during 72 h at RT and under Ar atmosphere. The bKGN was freeze-dried to obtain a white powder (30.5 mg, yield of 34 %). Both reactions were monitored by silica TLC (DCM-MeOH 9:1) and purified by preparative HPLC (Shimadzu, Kyoto, Japan) using a C18 column (5 μm bead particle size, Macherey-Nagel, Düren, Germany). The mobile phase was composed of 0.1 % (v/v) trifluoroacetic acid (TFA) in water (A) and acetonitrile (B). The following gradient elution was used for both reactional steps at a flow rate of 10 mL/min: 90 % B (0-5 min), 90-10 % B (5-45 min), and 10 % B (45-50 min). The intermediate product **1** and final compound were characterized by ^1^H NMR, ^13^C NMR, and HRMS (Figs. S1–S7).

### 2.3. Formulation of avidin-biotin-PEG_2_-kartogenin conjugate (Av-bKGN)

The bKGN was first dissolved in DMSO (1 mg/mL) and conjugated to avidin in PBS in a molar ratio of 4:1. The mix was vortexed at 600 RPM (Vortex-Genie^®^ 2 mixer) over 1 h at RT to obtain the avidin-biotin-PEG_2_-kartogenin (Av-bKGN) conjugate. The 2-(4′-hydroxyazobenzene) benzoic acid (HABA) assay was set up using the Pierce^™^Biotin Quantification Kit to determine the amount of bKGN necessary to form the Av-bKGN conjugate. The kit’s complex avidin-HABA (1.5 mg) was dissolved in 2 mL (0.75 mg/mL) of PBS pH 7.4 and equilibrated at RT under magnetic stirring. The starting absorbance was determined at 500 nm with a UV-vis spectrophotometer (Beckman Coulter^®^, USA). Then the bKGN was added to increase the molar ratio with respect to the avidin-HABA. After magnetic stirring for 1 min the solution was equilibrated at RT and the absorbance was assessed at 500 nm. A quartz cuvette was used for each measurement, and free KGN was used as a blank.

### 2.4. Physicochemical characterization of Av-bKGN

The zeta potential and the particle size distribution (PSD) of the Av-bKGN conjugate were measured in triplicate in PBS and simulated synovial fluid (SF) by the Zetasizer Nano ZS (Malvern, UK). For the size measurement, a semi-micro cuvette (BRAND^®^, Sigma Aldrich) was used, and 2 mg/mL of free protein or protein conjugate was filtered through a 0.22 μm filter (BGB^®^, China) before the measurement. The experimental size was compared to the theoretical diameter of 7.2 nm estimated by the Protein Utility tools (Malvern Zetasizer Nano software version 7.13, Malvern, UK), based on the Stokes-Einstein equation for globular protein, considering the 66 kDa molecular weight of Av. While the zeta potential measurement of the free protein and protein conjugate (2 mg/mL) in PBS 0.1x required a folded capillary zeta cell (Malvern, UK) to reach an optimal conductivity (<0.1 mS/cm).

The Asymmetric Flow Field-Flow Fractionation (AF4) method was employed to assess avidin-albumin interactions. Samples were diluted in PBS (5 mg/mL) and incubated at 37 °C during 1 h, under constant shaking. Separation was performed using a Postnova AF2000 AF4 system (Postnova Analytics, Germany) equipped with UV and multi-angle light scattering (MALS) detectors. The analysis was conducted over a total duration of 30 min. Injected sample volume was 100 μL. An amphiphilic membrane made of modified regenerated cellulose with a 10 kDa molecular weight cut-off was utilized. MilliQ water was used as the mobile phase, ensuring optimal separation. The specific flow rates and times for each step of the analysis are resumes as following. Focus step: injection flow 0.50 mL/min during 2 min, cross flow – constant 3 mL/min during 2 min, focus flow 3 mL/min during 0.5 min; elution step: constant cross flow 3 mL/min, linear cross flow 3-0 mL/min during 5 min, constant cross flow 0 mL/min during 5 min. Rinse step: tip/focus pump 0.1 mL/min during 0.5 min. To study potential interactions, elution times of each sample were monitored and compared, and the radius of gyration (Rg) was determined.

### 2.5. Stability study in simulated synovial liquid, physiological and esterase enriched medium

The stability of Av-bKGN was investigated over 24 h in simulated SF, in physiological saline (PBS, pH 7.4), and in the presence of porcine liver esterase (PLE). Av-bKGN was first formulated and diluted in 1 mL of SF, PBS pH 7.4, PBS with 1U PLE and PBS with 100U PLE at a final bKGN concentration of 9.5 μg/mL. The samples were incubated at 37 °C during 24 h and then filtered through a 0.22 μm filter in a UHPLC vial. The free drug was quantified using an Acquity^™^ Ultra-high performance liquid chromatography (UHPLC) coupled to a photodiode array (PDA) detector (Waters Corp, Milford, MA, USA). An Acquity UHPLC BEH C18 column (2.1 mm × 50 mm, 1.7 μm, Waters, USA) was equilibrated at 40 °C. The separation of 3 min was performed at a constant flow rate of 0.4 mL/min using a mobile phase composed of 0.1 % (v/v) formic acid in water (B) and acetonitrile (A) with gradient elution from 60 to 20 % A (0-2.50 min) and 20 % to 60 % (2.50-3 min). The UHPLC gradient was used to separate all compounds: bKGN (retention time tR:0.413min), KGN (tR:1.22min), and the highlighted 2-([1,1′-biphenyl]-4-yl)isoindoline-1,3-dione (ISO) (tR:2.16min) (Fig. S8). A first calibration curve was realized using the synthesized KGN described in our previous publication [27], ranged from 0.1 to 15 μg/mL with a linearity >0.99 (Fig. S9). A second calibration curve was realized using the 2-([1,1′-biphenyl]-4-yl)isoindoline-1,3-dione (ISO) synthesized in acidic medium ranged from 0.7 to 3 μg/mL with a linearity >0.99 (Fig. S10).

### 2.6. In vitro studies

#### 2.6.1. Cytotoxicity assays

Human chondrocytes (hCH) (PromoCell, Germany) and Human Mesenchymal Stem Cells from Bone Marrow (hMSC-BM) (PromoCell, Germany) were maintained in a cell medium supplemented with 10 % fetal bovine serum and 1 % penicillin/streptomycin. The cell culture flasks were incubated at 37 °C, with 5 % carbon dioxide in a humidified atmosphere. Experiments were performed from passage 2 until 4. For the *in vitro* mitochondrial activity assay, hCH cells, and hMSC-BM were seeded on 96-well plates at a density of 20,000 cells per 100 μL/well. After 24 h, Av-bKGN dissolved at 1 mg/mL in DMSO were added into the cell medium in triplicate wells at final concentrations ranging from 0.1 to 20 μM. After 24 h of incubation, 10 μL of the cell proliferation reagent WST-1 (Abcam, USA) was added to each well. Absorbance was measured 2 h later using a Synergy Mx microplate reader (BioTek Inc., USA) after 1 min of shaking at a test wavelength of 450 nm and a reference wavelength of 650 nm. The mitochondrial activity was normalized and expressed in percentage. In addition, a Lactate Dehydrogenase (LDH) assay was realized in the same hCH conditions, using the LDH-Glo^™^ Cytotoxicity Assay (Promega, USA) and the cytotoxicity was expressed in percentage.

#### 2.6.2. NO assay in macrophages cell line

Macrophages cell line (RAW 264.7) (ATCC, USA) were incubated in a cell culture flask supplemented with DMEM/F-12 (Gibco, USA), 10 % heat-inactivated fetal bovine serum and 1 % penicillin/streptomycin at 37 °C with 5 % carbon dioxide in a humidified atmosphere. The experiments were performed in a 96-well plate with 50,000 cells per 100 μL/well in a serum-free medium. After 24 h, 100 ng/mL of lipopolysaccharide (LPS) from *S. minnesota* R595 (Inaxon, UK) were introduced into wells, excepted for the control. The inflammation was induced during 24 h and 1 μM of bKGN, ISO and iNOS were finally added to the plate. The Griess assay (Promega, USA) was performed after 24 h of cells treatment to quantify the residual nitrites (NO^−^_2_). Cellular experiments were performed from passage 9 until 11 in triplicate. Absorbance was assessed within 30 min in a microplate reader (Synergy Mx microplate reader, BioTek Inc., USA) at a wavelength of 530 nm. A nitrite calibration curve ranging from 1.5 to 100 μM was realized in triplicate with a linearity >0.99 (Fig. S13).

#### 2.6.3. IL-6 immunoassay in hCH culture

hCH were maintained in a cell medium supplemented with 10 % fetal bovine serum and 1 % penicillin/streptomycin. The cell culture flask was incubated at 37 °C, with 5 % CO_2_ in a humidified atmosphere. Experiments were performed from passage 10 to 13. hCH were seeded on 96-well plates at a density of 20,000 cells per 100 μL/well. Then the medium was replaced, and the inflammation was induced for 24 h with 1 ng/mL of IL-1β. Afterwards the cells were treated with 1 μM of Av-bKGN during 24 h. Finally, the Lumit^™^ IL-6 (Human) Immunoassay (Promega, USA) was performed in a 96 black well plate (Costar^®^, USA) using the supernatant, quantifying the luminescence correlated to the IL-6 released in the medium. A calibration curve was realized from 60.8 pg/mL to 100,000 to of IL-6 with a linearity >0.99 (Fig. S14).

### 2.7. Ex vivo experiments

#### 2.7.1. Articular cartilage harvest

Fresh 1-3 weeks old bovine calf knee joints were provided by a local butcher and cleaned using a n° 10-blade scalpel to expose articular cartilage from the patellofemoral groove. Fat, muscles, ligaments, tendons, and any other connective tissue were removed. Using 3-mm and 6-mm dermal punches, perpendicular punches were made into the cartilage as described previously [28]. Cylindrical explants were removed and placed into individual wells of a 48-well plate containing 500 μL of 1x PBS supplemented with 1 % v/v PSA. The cartilage explants were then sliced using a razor blade and a cutting device to obtain a 1-mm thick cartilage explant, including the superficial zone [26]. The procedure was repeated for each explant to obtain homogeneous samples of 1 mm thickness and 3 mm/6 mm diameter. The 4 mm/diameter explants were harvested using 4 mm dermal punches, with an unchanged thickness of 2 mm. The cartilage explants were stored individually in polypropylene tubes containing 500 μL of 1x PBS supplemented with protease inhibitors (PBS-PI, 1 PI mini-tablet per 50 mL of 1x PBS) at −20 °C. Before each of the following experiments, the tubes containing the explants were thawed for 30 min in a 37 °C water bath.

#### 2.7.2. Cartilage uptake ratio (Ru)

The cartilage uptake of Av-bKGN through 4 mm diameter and 2 mm thickness bovine explants was investigated by fluorescence in a 48-well plate. Avidin (Av) and its neutral counterpart neutravidin (Ne) were conjugated to biotin (5-fluorescein) (b5) and bKGN in a molar ratio of 1:1:3 to minimize the amount of fluorophore present in the nanostructure. The samples Av - b5/bKGN and Ne - b5/bKGN were vortexed for 1 h at 600 RPM to form the tetrameric conjugates. The initial fluorescence at time 0 (converted into initial concentration *C_Bath_,_i_*) of Av - b5/bKGN, Ne - b5/bKGN, and free b5 was measured in 500 μL of PBS and simulated SF in a 48-well plate (Costar^®^, USA) using a SynergyMx plate reader (BioTek^®^, GER). The cartilage disks were introduced into the wells and incubated at 37 °C for 24 h. Then, the disks were removed from the plate and the remaining fluorescence (converted into *C_Bath_*) measured. The amount of Av - b5/bKGN, Ne - b5/bKGN, and b5 uptake by cartilage was expressed as the uptake ratio *R_u_* and calculated using the following formulae [29]:

$$C_{\text{Cartilage}}=\frac{\left(C_{\text{Bath},i}-C_{\text{Bath}}\right)\;\text{x}\;V_{\text{Bath}}}{\text{Tissue Wet Weight}}\left[\frac{(\mu M-\mu M)\;x\;\mu L}{mg}\right]$$

$$R_{u}=\frac{C_{\text{Cartilage}}}{C_{\text{Bath}}}$$

The fluorescence was converted to concentrations using the corresponding calibration curves, which ranged from 0.00315 to 1 mg/mL with a linearity >0.99 for b5, Av-b5/bKGN and Ne-b5/bKGN (Figs. S15–17). The excitation/emission wavelengths of b5 (481 nm/550 nm) were used for all fluorescent measurements.

#### 2.7.3. Cartilage retention over 10 days

The bovine cartilage cumulative retention of Av - b5/bKGN, Ne - b5/bKGN, and free b5 were monitored) over 10 days using fluorescence. The starting fluorescence for the 3 samples was first assessed on a 48-well plate. Every 24 h of incubation at 37 °C the disks were removed and placed into a new well containing 500 μL of PBS or simulated SF and the remaining fluorescence was measured. The cumulative cartilage retention expressed as a percentage over 10 days was calculated using the above-mentioned calibration curves.

#### 2.7.4. Real-time measurement of one-dimensional diffusion

**Fig. 2.**
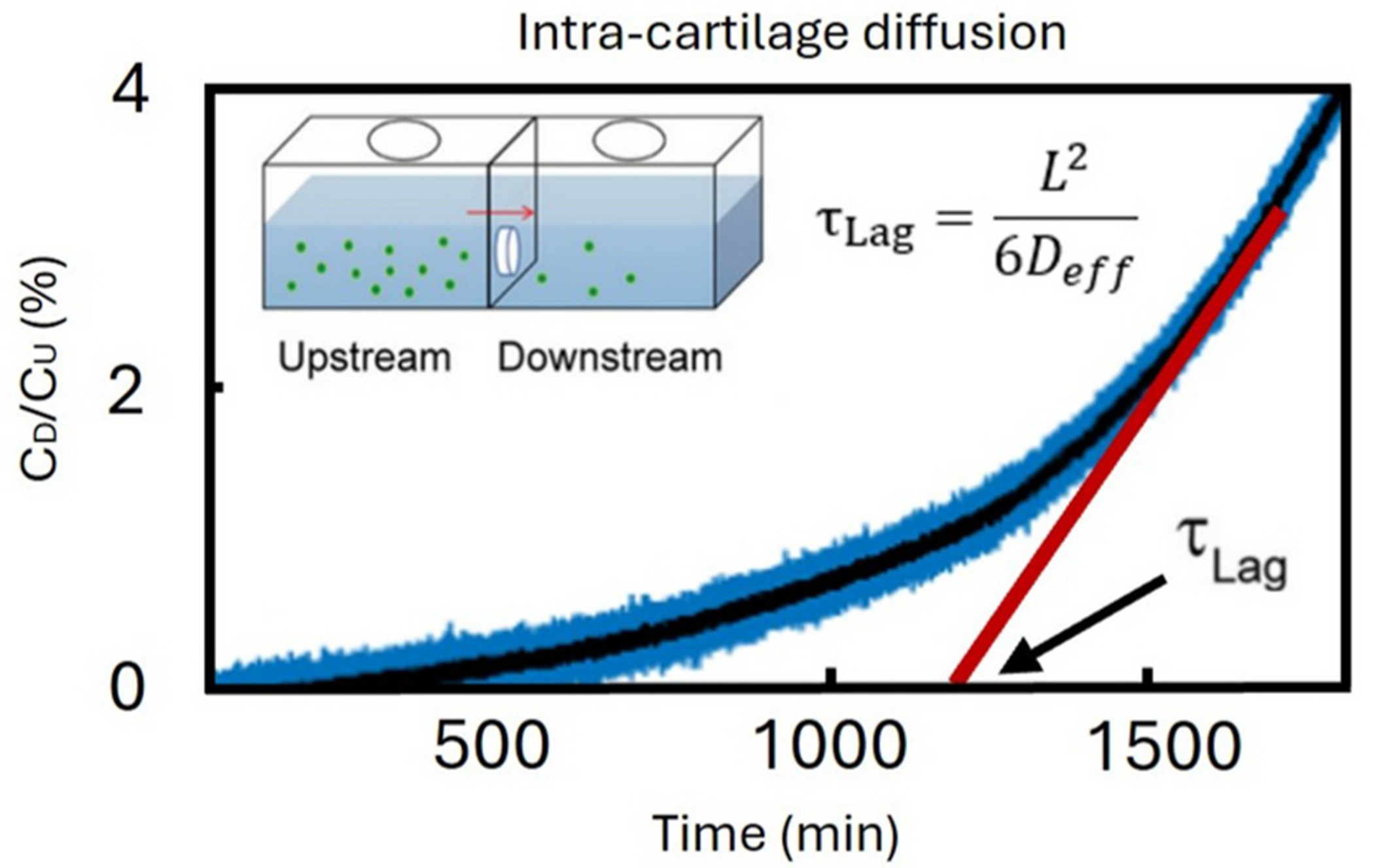
Real-time measurement of non-equilibrium diffusion curve in cartilage. L corresponds to cartilage thickness measured at each experiment, and τ_Lag_ is the time at which the nanocarrier-b5 reaches a steady state flux. τ_Lag_ was estimated using the time-axis intercept of the linear slope of the normalized concentration versus time. Adapted from Ref. [30].

The real-time one-dimensional diffusion of the avidin-based delivery system through cartilage disks of 6 mm diameter was investigated using a poly (methyl methacrylate) transport chamber developed by A. G. Bajpayee et *al*. [19,26]. First, the two compartments were equilibrated with 0.5 % non-fat-dried bovine milk solution in PBS for 15 min to prevent non-specific adsorption of solutes to the inner walls of the transport chamber. Each compartment was then washed two times with 2 mL PBS. The cartilage disk was then clamped by O-rings between the two compartments of the diffusion chamber with the superficial zone exposed to the upper chamber, with an exposed tissue area for transport of 0.126 cm^2^/disk. The two chambers were assembled, and each compartment was filled with 2 mL of PBS-PI. The blank fluorescence was equilibrated in real time over 5 min. At the starting time t = 0 s, the samples (b5, Av-b5 (1:4), Av-b5/bKGN (1:1:3) and Ne-b5 (1:4)) were added to the upstream compartment resulting in a one-dimensional transport through the cartilage tissue into the downstream compartment. The baths in both compartments were magnetically stirred to minimize the effects of stagnant layers at the solution-tissue interfaces. The concentration of the fluorescently labelled nanocarrier in the downstream chamber was measured in real time by exciting the downstream solution using a spectrofluorometer 488 nm laser and detecting the emission. Non-equilibrium diffusion curves were thereby obtained from real-time fluorescence of downstream chamber by plotting normalized downstream concentration (C_D_) to upstream concentration (C_U_) over time as shown in Fig. 2. The ratio was expressed in percentage (R = C_D_/C_U_) as a function of time. By extrapolation at the steady state, the time necessary to reach the steady state flux (*τ_Lag_*) was foundand the effective diffusivity of the nanocarriers was calculated (D_eff_) [15] (Fig. 2).

#### 2.7.5. GAGs loss quantification in OA cartilage explant cultures

**Fig. 3.**
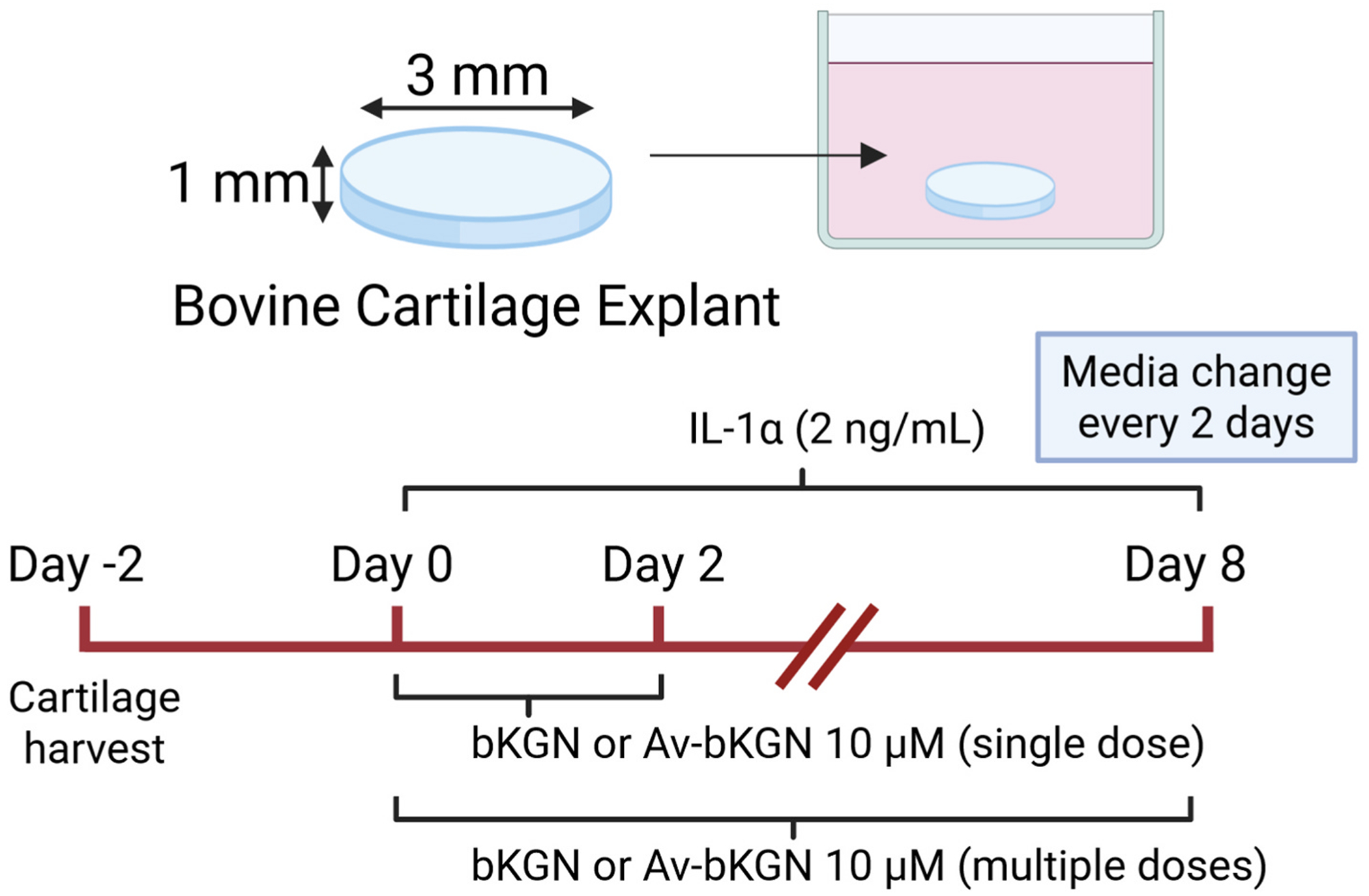
Schematic of the experimental design of GAGs loss quantification in the OA cartilage explant cultures.

Fresh 3-mm diameter cartilage explants harvested from calf knee joints were equilibrated in a 96-well plate culture medium containing 96.2 % low glucose DMEM, 1.0 % HEPES buffer, supplemented with 1.0 % PSA, 1.0 % ITS, 1.0 % NEAA, 0.4 % ascorbic acid and 0.4 % proline for 48 h at 37 °C and 5 % CO_2_ for 2 days before treatment. To test the chondroprotective properties of the Av-bKGN nanocarrier, equilibrated cartilage explants were treated with or without IL-1α (2 ng/mL) for 8 days in combination with i) a single dose of bKGN (10 μM), ii) a single dose of Av-bKGN (1:4) (10 μM of bKGN), iii) multiple doses of bKGN (10 μM) and iv) multiple doses of Av-bKGN (1:4) (10 μM of bKGN). Every 2 days the media was changed and IL-1α was replenished at each medium change as shown in the schematic of the experimental design (Fig. 3) based on the previous work [14].

The concentration of IL-1α it was selected to represent a moderately aggressive cytokine treatment [31]. Explants treated with a single dose were exposed to bKGN and its Av-carrier for the first 2 days only; in subsequent media changes the media did not contain the drug, simulating a single intra-articular injection *in vivo* [15]. In the continuous dosing condition, bKGN and Av-bKGN were replenished every 2 days throughout the culture period. The single dose of Av-bKGN was compared to the multiple doses of free drug (bKGN) to highlight the importance of the drug depot-forming capability of the Av-based carrier. After 8 days of culture, each cartilage explants were weighed and digested in proteinase K/Tris buffer solution during 48h at 57 °C. The GAGs released in the media at each time point and the residual GAGs in the digested explants were then measured using the 1,9-dimethylmethylene blue (DMMB) assay. [32]. For the quantification of GAGs in the supernatants and digested explants, calibration curves were made with chondroitin sulfate as shown in Fig. S18 with a linearity >0.99. The DMMB assay is based on the formation of a purple complex between the dimethyl methylene blue dye and the GAGs components of cartilage matrix quantified at 525 nm. The cumulative GAG loss percentage was calculated by normalising the cumulative GAG loss in the supernatant to the total GAGs, (sum of the residual GAG in the digested explants and the total GAG released over the course of the eight-day experiment).

### 2.8. Statistical analysis

For cartilage uptake experiments *n* = 5 independent explants per condition coming from the same animal donor. Statistical comparison between different groups was performed using an ANOVA One-Way analysis (Tukey’s multiple comparison test) with GraphPad Prism 8.0.1 software (San Diego, CA, USA). The same statistical analysis was performed for the *in vitro* assays (NO^−^_2_ and IL-6), with *n* = 3. Cartilage retention study over 10 days were conducted using *n* = 5 independent explants per condition coming from the same animal donor and statistical significances were obtained through ANOVA Two-Way analysis followed by Dunnet multiple comparison test to the Ne group. The same statistical analysis was performed for the GAG loss assay, with *n* = 5 independent explants coming from at least three animal donors and IL-1α serving as the comparative group. Data were reported as the mean ± s.d., considering a 95 % confidence interval at significance level *p < 0.05, **p < 0.01, ***p < 0.001, and ****p < 0.0001.

## 3. Results

### 3.1. 2-Steps synthesis of bKGN

The intermediate product 1 and the final product bKGN were synthetized successfully with a yield of 88 % and 34 %, respectively. This 2-step protocol makes bKGN synthesis easily scalable to large quantities. The ^1^H NMR, ^13^C NMR, and HRMS spectra confirmed both structures (Figure. S1 to Figure.S7).

Intermediate product 1: ^1^H NMR (600 MHz, DMSO-*d_6_*) δ 13.26 (s, 1H), 7.80-7.82 (m, 1H), 7.78-7.76 (m, 1H), 7.76-7.63 (m,3H), 6.41 (s, 1H), 6.35 (s, 1H), 4.34-4.29 (m,3H), 4.13-4.11 (m, 1H), 3.70-3.68 (m, 2H), 3.45-3.43 (m, 2H), 3.36 (H2O), 3.22-3.19 (m,2H), 3.10-3.07 (s, 1H), 2.83-2.80 (s, 1H), 2.51 (DMSO), 2.59-2.57 (m, 1H), 2.08-2.03 (m, 2H), 1.63-1.57 (m,1H), 1.53-1.42 (m,3H), 1.43-1.25 (m,2H). ^13^C NMR (151 MHz, DMSO-*d_6_*) δ 172.71, 168.48, 167.99, 163.20, 132.72, 132.56, 131.82, 131.63, 129.33, 128.70, 69.57, 68.24, 64.80, 61.50, 59.66, 55.88, 38.85, 35.55, 28.64, 28.47, 25.70. HRMS ESI+: *m*/*z* 502.176 [M+Na]^+^ (calculated for C_22_H_29_N_3_O_7_S 502.15). HRMS ESI-: *m*/*z* 478.176 [M – H]^−^ (calculated for C_22_H_29_N_3_O_7_S 478.17).

bKGN: 1H NMR (600 MHz, DMSO-*d6*) δ 10.53 (s, 1H), 7.91-7.89 (m, 1H), 7.82-7.80 (m, 2H), 7.78-7.76 (m,1H), 7.74-7.72 (m,1H), 7.68-7.63 (m, 5H), 7.48-7.45 (m, 2H), 7.36-7.33 (m,1H), 6.40 (s, 1H), 6.35 (s, 1H), 4.30-4.28 (m, 3H), 4.12- 4.09 (m, 1H), 3.60-3.58 (m, 2H), 3.34 (H2O), 3.14-3.11 (m, 2H), 3.08-3.05 (m, 1H), 2.82-2.79 (m, 1H), 2.63-2.61 (m, 1H), 2.51 (DMSO), 2.40-2.39 (m, 1H), 2.09-2.07 (m, 2H), 2.03-2.01 (m, 2H), 1.61-1.55 (m, 1H), 1.49-1.40 (m,3H), 1.31-1.22 (m,2H). ^13^C NMR (151 MHz, DMSO-*d_6_*) δ 172.60, 167.43, 166.61, 163.18, 140.23, 139.31, 139.07, 135.63, 132.67, 130.23, 129.92, 129.38, 128.45, 127.52, 127.34, 126.76, 120.38, 69.55, 68.26, 64.77, 61.50, 59.66, 55.88, 46.36, 46.33, 38.76, 36.25, 35.52, 31.24, 28.66, 28.48, 26.40, 26.35, 25.69. HRMS ESI+: *m*/*z* 631.257 [M+H]^+^, 653.242 [M+Na]^+^ (calculated for C_34_H_38_N_4_O_6_S 631.25 and 653.23).

### 3.2. Formulation of Av-bKGN and its physicochemical characterization

**Fig. 4.**
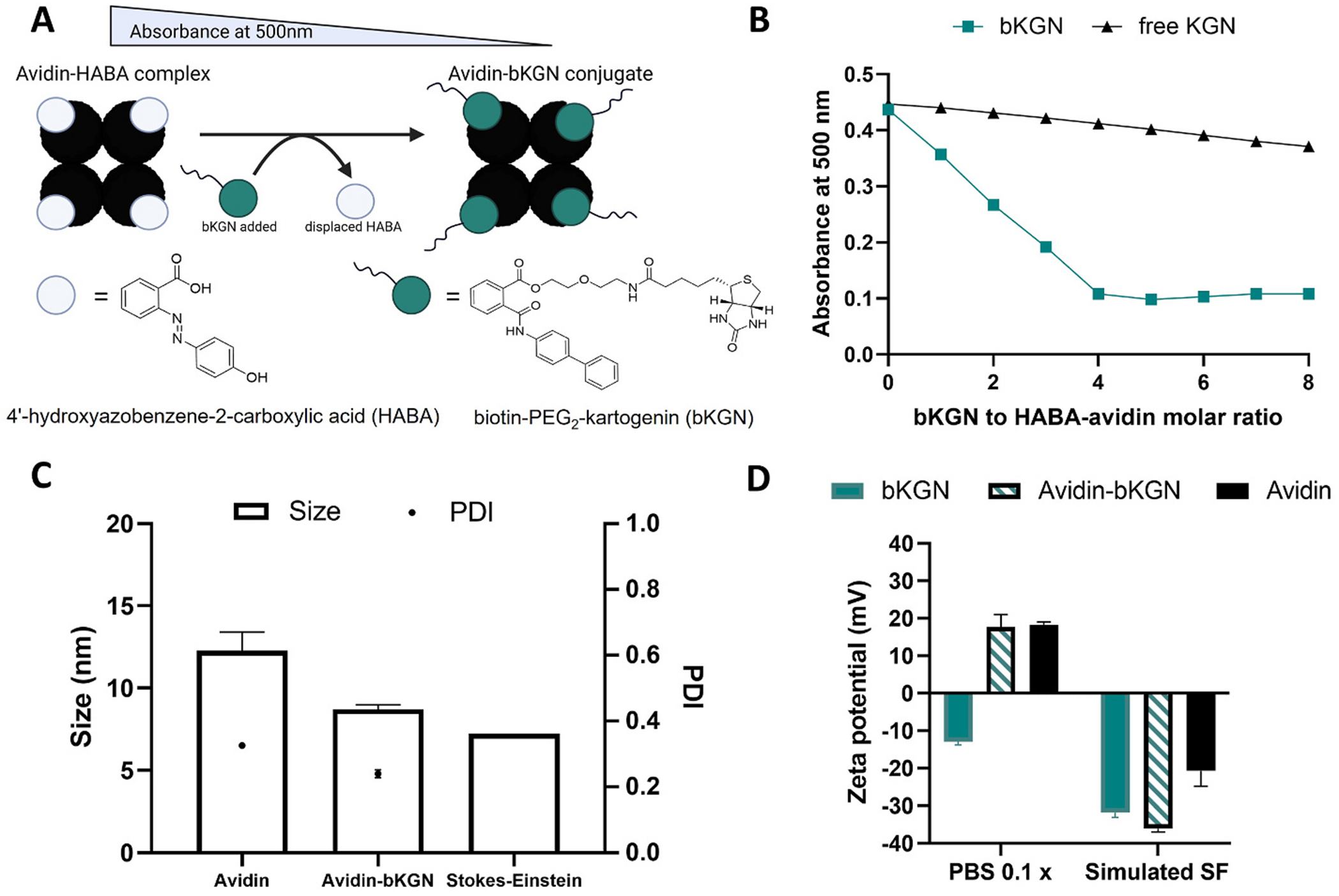
(A) Schematic representation of the formation of the tetrameric Av-bKGN; the bKGN displaces the HABA from the binding site of the avidin enabling the conjugate formulation. (B) HABA assay performed with bKGN and free KGN at 500 nm. (C) Average size and polydispersity determined by laser diffraction, compared to the theoretical size calculated using the Stokes-Einstein equation for globular proteins. (D) Zeta potential values measured in PBS and simulated SF. The results (C-D) are presented as the mean ± sd (*n* ≥ 3).

Appropriate formulation of the Av-bKGN was assessed with the HABA assay. The HABA assay is based on the following principle: 4 M ratios of bKGN displace the HABA and form the tetrameric avidin conjugate (Fig. 4A). Indeed, a plateau is reached at a molar ratio of 4 bKGN to HABA-avidin (Fig. 4B), confirming the assembly of the tetrameric structure. In contrast, the free KGN used as blank showed a slight decrease in term of absorbance at 500 nm indicating a negligible binding. The size of the tetrameric globular Av-bKGN measured in physiological medium by dynamic light scattering was 8.7 ± 0.26 nm (PDI of 0.24), close to the theoretical one estimated by Stokes-Einstein equation of 7.22 nm. The free Av show higher size of 12.26 ± 1.15 nm and PDI of 0.32 (Fig. 4C). The negative zeta potential of bKGN in PBS (−12.9 ± 0.87 mV) was reversed to a positive one after conjugation to Av (18.3 ± 0.75 mV), a value close to that of the free protein (17.6 ± 3.37 mV). In simulated SF, all samples (bKGN, Av-bKGN and Av) had a negative charge (− 31.8 ± 1.32 mV, −36.1 ± 0.80 mV and −20.7 ± 4.13 mV) (Fig. 4D), probably due to albumin or hyaluronic acid coating, since these molecules are highly common in the simulated SF. The AF4 fractograms (Fig. S19) of avidin, albumin and avidin + albumin signal from UV-MALS reveals that the radius of gyration (Rg) was 6.9 nm for avidin and 9.3 nm for albumin. As avidin is smaller than albumin, the difference in their separation behaviour is sufficient for assessing interaction. The mixing of albumin and avidin produced a specific elution peak at around 6.5 min with Rg value of 14.6 nm demonstrating an interaction between the two proteins.

### 3.3. Identification of kartogenin isoindoline derivative in SF, PBS and esterase enriched medium

**Fig. 5.**
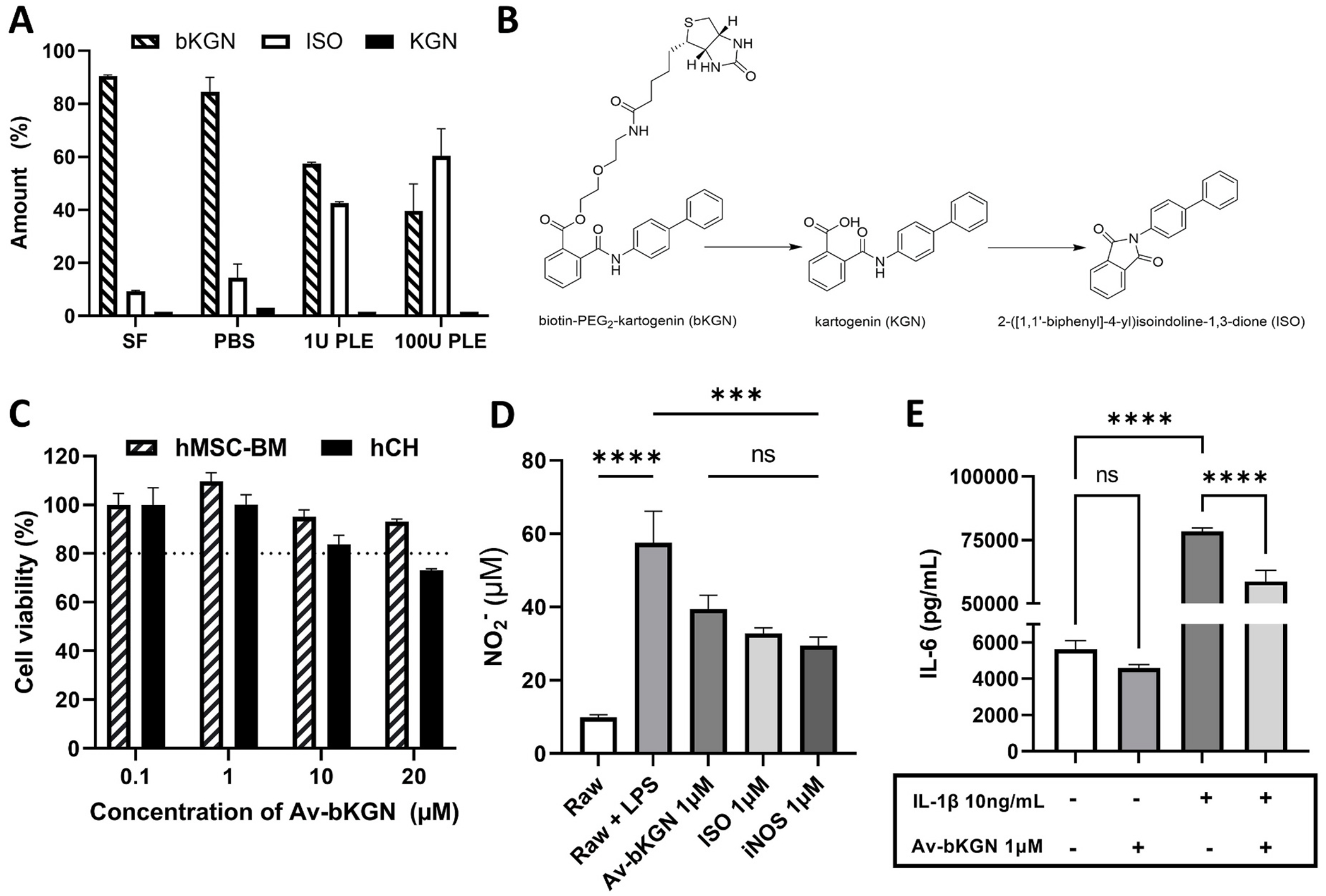
(A) Stability study of Av-bKGN in SF, PBS pH 7.4 and in presence of 1 or 100 units of PLE at 24 h. (B) Schematic representation of the ISO derivative compound formation. (C) Cell viability of hCH and hMSC-BM after treatment of Av-bKGN in a concentration range from 0.1 to 20 μM. (D) Detection of nitrite/NO^−^_2_ (μM) released in the medium from macrophage cell line Raw. 264.7 after inflammation with LPS, and treatment with 1 μM of Av-bKGN, ISO or iNOS. (E) IL-6 (pg/mL) detected after inflammation with 10 ng/mL of IL-1β and treatment with 1 μM of Av-bKGN. The results are presented as mean ± sd (*n* = 6 for A, *n* = 3 for C-E). ***p < 0.001, ****p < 0.0001 (ANOVA One-Way analysis followed by Tukey’s multiple comparison test). ns means non-significant.

After 24 h of incubation of Av-bKGN in PBS and SF, around 84 % and 90 % w/w of bKGN remained stable, unchanged, while a new structure appeared corresponding to an isoindoline derivative compound (ISO). Relative amounts of 13 % w/w of ISO and around 1.5 % w/w of KGN were quantified in PBS medium while 10 % of ISO and no KGN was found in SF after 24 h of incubation (Fig. 5A). In presence of 1U and 100U of PLE the amount of ISO increased by up to 40 to 60 % due to the faster enzymatic kinetics, while the amount of bKGN decrease. No KGN was detected in presence of 1U and 100U PLE, probably due to the fast and complete cyclisation of KGN into the ISO derivative compound represented in Fig. 5B. The identification of the ISO structure was confirmed using the calibration curve of the ISO synthesized (Fig. S10) having same elution time (tR: 2.16min). ISO present different retention time than the precursor 4-aminobiphenyl (tR: 0.79) as shown in Fig. S11.

### 3.4. In vitro studies

#### 3.4.1. Cytotoxicity assay

The mitochondrial activity of hCH and hMSC-BM remained above 80% up to a concentration of 10 μM Av-bKGN, with low variability between cells, and decreased slightly at higher concentrations (Fig. 5C). At 20 μM the mitochondrial activity of hCH was below 80 %, which could be characterized as “toxic”. However, LDH release test (Fig. S12) did not show significant enzyme leakage compared to the untreated control, suggesting that the cells had a lower mitochondrial activity, but were not damaged by the compound.

#### 3.4.2. Av-bKGN reduces inflammatory mediators in vitro

The anti-inflammatory properties of Av-bKGN and ISO were investigated performing a Griess assay. Macrophages strongly inflamed with LPS released around 6-fold more nitrites in the media (Fig. 5D). After 24 h of treatment with 1 μM Av-bKGN, ISO or iNOS, the concentration of NO^−^2 decreased significantly, from 57 ± 8 % in the LPS-induced inflammation groups to 34 ± 5 % in the treated groups. No significant difference was observed between the three treatment groups. This is the first study in which a biological activity of the ISO derivative, synthesized as shown in Fig. S10, was tested *in vitro*. bKGN as well as ISO presented in their structure the 4-aminobiphenyl, for which an anti-OA activity was reported previously in the literature [33].

The IL-6 released by inflamed hCH in the media was quantified by immunoassay (Fig. 5E). After 24 h of IL-1β inflammation, the amount of IL-6 released increased 12.5-fold, from around 6000 to 75,000 pg/mL. By treating hCH with 1 μM of Av-bKGN during 24 h, the amount of IL-6 was reduced by 25 %.

### 3.5. Ex vivo experiments

#### 3.5.1. Av-bKGN has an enhanced uptake and retention in bovine cartilage explant

**Fig. 6.**
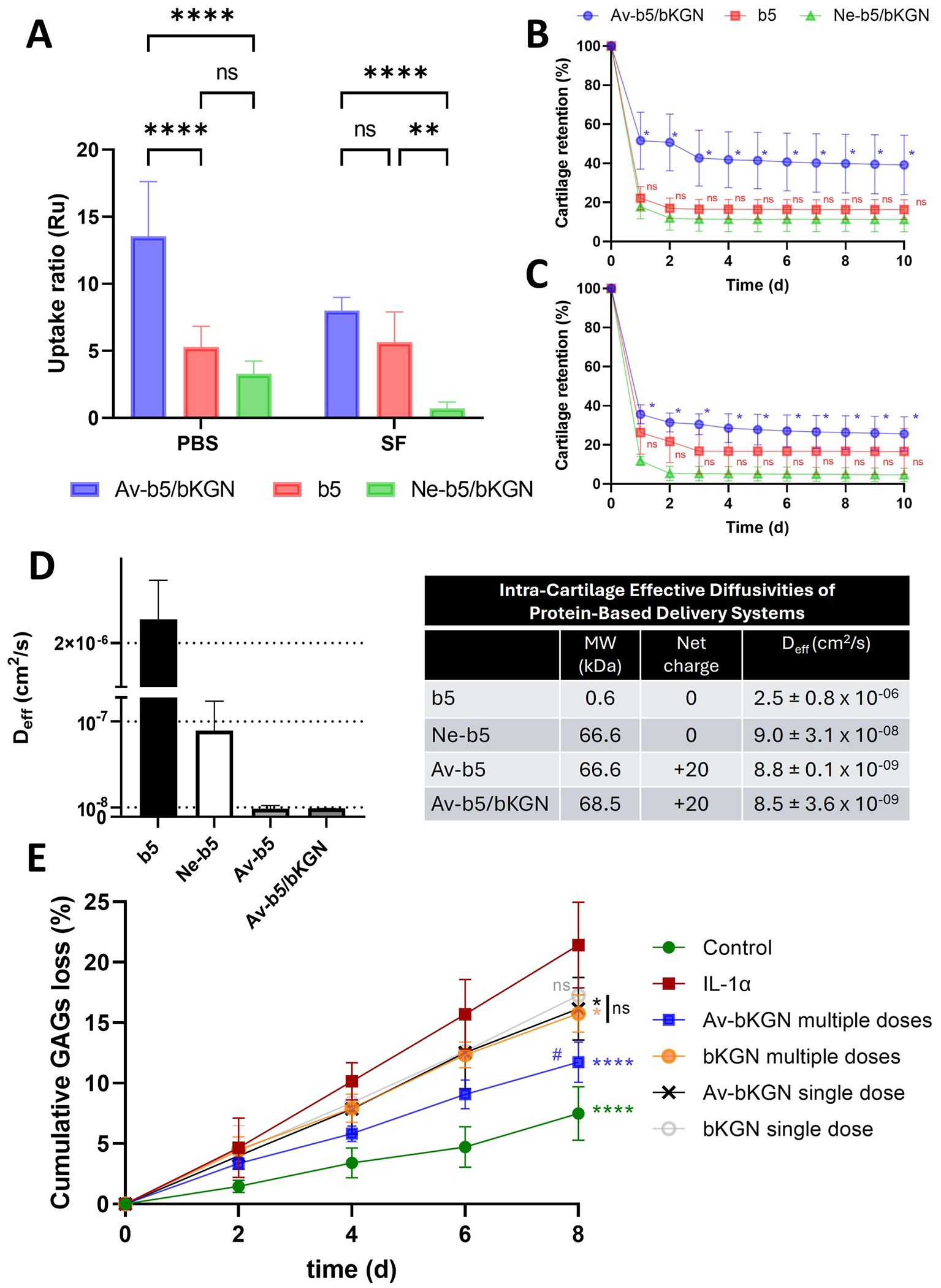
Cartilage explant experiment of protein-based delivery systems. (A) Uptake ratio of protein-based delivery systems in cartilage explant over 24 h in the presence of PBS or simulated SF. Intra-cartilage retention of protein-based delivery systems over 10 days in (B) PBS or (C) simulated SF. (D) Effective diffusivity (D_eff_) of protein-based delivery systems through cartilage explants in PBS. (E) Cumulative GAGs loss after single dose of bKGN *vs*. multiple doses of bKGN *vs*. multiple doses of Av-bKGN *vs*. single dose of Av-bKGN in IL-1-stimulated OA model. Results are represented by the mean ± sd (*n* = 5 for all results, except for D *n* = 3). *p < 0.05, **p < 0.01, ***p < 0.001, ****p < 0.0001 (ANOVA One-Way analysis followed by Tukey’s multiple comparison test) for A; *p < 0.05 (ANOVA Two-Way analysis followed by Dunnet multiple comparison test to the Ne-b5/bKGN group) for B and C. *p < 0.05, **p < 0.01, ***p < 0.001 (ANOVA Two-Way analysis followed by Dunnet multiple comparison test: * *vs*. IL-6 group, # *vs*. bKGN p < 0.05). ns means non-significant.

It was shown that Avidin-based system (Av-b5/bKGN) exhibited the highest uptake ratio of ~14X following 24 h of equilibrium uptake in PBS (Fig. 6A). This is much higher than the uptake ratio of the neutral Ne-b5/bKGN and b5 (Ru = ~3X and ~5x, respectively). The elevated uptake of Av-b5/bKGN highlights the effect of electrostatic attraction between the compound and the negatively charged cartilage matrix to enhance the uptake in the tissue. In the presence of simulated SF, the intra-cartilage uptake of all compounds reduced significantly. Av-b5/ bKGN uptake ratio was reduced almost by half (Ru = ~8X) and Ne-b5/bKGN by four (Ru = 0.7X). This is expected as the simulated SF contains albumin, hyaluronic acid, nutrients, and ions which can interact with the avidin compound prior to uptake and hindered its intra-cartilage uptake. Meanwhile, no significant uptake alteration was observed for free dye b5 uptake due to the introduction of simulated SF (Ru = ~5X).

The explant retention over 10 days in PBS displayed in Fig. 6B shows an average value of ~40 % for the Av-b5/bKGN system compared to around ~20 %-a 2-fold reduction-for both Ne-b5/bKGN and b5. This highlights the role of electrostatic interaction which help the retention of cationic compound within the anionic cartilage matrix. In the presence of SF, Av-b5/bKGN intra-cartilage retention were reduced to ~30 %. This was expected since the presence of SF constituents provide more interactions that could desorb more of the uptaken compound out of the matrix. In both PBS and SF, Av-b5/bKGN exhibited significant (p *<* 0.05) increase in intra-cartilage retention compared to their neutral counterparts. Interestingly, there was no significant difference in intracartilage retention between Ne-b5/bKGN and b5 in either condition (PBS or SF; see Fig. 6B and C). However, as shown in Fig. 6C, the trend for cartilage retention was lower for the Neb5/bKGN than for free b5 in SF.

#### 3.5.2. Electrostatic interactions slow Av diffusivity through cartilage matrix

Av-b5 exhibited an effective diffusivity of ~8 × 10^−9^ cm^2^/s through 6 mm-cartilage explant, which is ~1000–4,000x slower than Ne-b5 and b5, suggesting its strong binding interaction with the cartilage matrix. The D_eff_ value remained similar around ~8 × 10^−9^ cm^2^/s when the drug (bKGN) was conjugated to the protein, demonstrating minor effect of the conjugation on transport properties. Ne-b5 exhibited a 1-order magnitude faster Deff than Av-b5 which can be attributed to its weaker binding with cartilage matrix.

#### 3.5.3. Av-bKGN ensure chondroprotection of cartilage matrix

Av-based delivery system of KGN showed promising cartilage transport properties which could lead to an effective biological efficacy for OA treatment. Thus, we tested its biological efficacy to rescue cartilage matrix loss in an *ex vivo* cytokine-induced OA model based on bovine cartilage explant. In a dose-response study, continuous dose of Av-bKGN was more effective in suppressing the IL-1α-induced GAG loss from cartilage explant compared to the continuous dose of free bKGN (Fig. 6E). Both continuous treatment of Av-KGN and bKGN resulted in a significantly lower GAG loss compared to IL-1α only treatment (~12 % and ~16 % by day 8, respectively). However, both failed to suppress the GAG loss down to the control level (7.5 % by day 8). As a proof of concept for the use of Av-bKGN as a drug depot for one-time administration, a single dose of either Av-bKGN or free bKGN at 10 μM concentration were tested. A single dose of Av-bKGN significantly suppressed the GAG loss from the explant at a similar level as a continuous dose of bKGN (~16 %) (Fig. 6E). While the GAG loss did not reach the control level, these results showed the potential of an Av-based drug delivery system to enable a less frequent administration of KGN for OA treatment.

## 4. Discussion

Charge-based drug delivery system is a promising approach to deliver payload to challenging tissue such as cartilage [17,34]. The electrostatic attraction between the positively charged delivery system to the anionic cartilage matrix, owing to its high density of chondroitin sulfate-GAGs, facilitates the enhanced uptake of various cationic particle [21]. One of them is avidin (Av), a highly charged glycoprotein which has been previously investigated in several works on drug delivery to cartilage [15,20,30,35–37]. In this study, bKGN, a proanabolic DMOAD, was successfully synthesized via biotinylation in a scalable, two-step process and its structure confirmed by ^1^H NMR, ^13^C NMR, and HRMS spectra (Figure S1 to Figure S7). Biotinylation was selected as avidin-biotin is one of the strongest interactions in nature [23]. As a linker, we used a PEG2, based on our initial hypothesis and supported by the literature, that PEGylated surfaces on nanoparticles would hinder the ester bond and physically block enzymatic access [38]. This would prevent esterase activity due to steric hindrance.

The formation of the tetrameric conjugate Av-bKGN was confirmed by the complete displacement of 2-(4′-hydroxyazobenzene)benzoic acid from the avidin binding site at a molar ratio of 4:1, as demonstrated by the HABA assay (Fig. 4B). The limited porosity of the cartilage matrix (10 nm) and its dense, anionic, collagen/GAGs-rich composition make it challenging for conventional nanoparticles to penetrate cartilage [17, 39]. Av-bKGN physicochemical characterisation by DLS confirms the nanometre-scale dimensions (~7 nm) and positive charge (around +20 mV) of Av-bKGN, which are key parameters for cartilage-targeted drug delivery systems. These values are similar to those reported by He et *al.* [15]. The Av charge was reversed to −35 mV in simulated SF, probably due to nanoparticle−s biocorona. Indeed, it has already been demonstrated that biocoronas, which include adsorbed albumin and globulin can significantly affect nanoparticle behaviour in SF [40,41]. Results from AF4 (Fig. S19) showed an increased elution time, while Rg value increased to 14.6 nm when avidin and albumin were mixed, most probably due to hydrophobic interactions between the two proteins. Accordingly, Vedadghavami A. et *al.* [41] reported that avidin most likely interacts with albumin and globulins as cationic peptides via hydrophobic interactions. Although Av-bKGN acquires a net negative surface charge in simulated SF, likely due to adsorption of anionic components such as albumin, hyaluronic acid, and lubricin fragments, enhanced cartilage uptake and retention can nevertheless persist through several non-mutually exclusive mechanisms. These include (i) heterogeneous or “patchy” surface charge distributions, whereby localized cationic domains remain accessible despite a negative ensemble-averaged ζ-potential [42,43]; (ii) dynamic corona equilibria and changes when exposed to negatively charged surfaces and (iii) specific, reversible non-electrostatic affinity interactions, such as hydrogen bonding or hydrophobic interactions between SF-derived corona components and cartilage matrix components such as proteoglycans and collagen fibers (e.g., type II) [44].

With respect to KGN nanocarrier stability and biodegradation, Av-bKGN remained stable at over 80 % in PBS and simulated SF while in presence of porcine esterase a new isoindoline derivative -ISO- was identified as the sole degradation product. This is the first study to identify ISO as new metabolite of KGN. The intracyclisation of bKGN to ISO was favoured by the slightly acidic conditions (pH below 7) of the esterase-enriched medium. It is important to consider the esterases presence and activity in synovial fluid, and that their level in OA joints can increase due to inflammation [25,45]. The overexpression of esterases in the synovial joint during OA can impact the pharmacokinetic of free DMOAD as well as those chemically bound to delivery systems/nanocarriers, and influence their therapeutic output [24].

The structure of the new ISO compound was confirmed by ESI-MS, ^1^H-RMN and ^13^C-RMN in our previous work [27] and its different retention time to the precursor 4-aminobiphenyl (tR: 0.79 min *vs* tR: 2.16 min), confirms its nature as a new degradation product. The NO inhibition in macrophages suggests a bioactivity, to be confirmed in the context of OA *ex vivo* and *in vivo models*. Further pharmacokinetic and biodistribution studies are required for this new compound, whose physicochemical properties differ from those of KGN and the precursor 4-aminobiphenyl [33].

Although the WST-1 assay showed Av-bKGN to slow down total mitochondrial activity at concentrations over 20 μM, the LDH assay did not detect any toxicity. In fact, it is important to assess cytotoxicity using multiple cell assays since WST-1 only provides an indirect indication of toxicity. LDH, on the other hand, is directly related to cell membrane damage and therefore provides more reliable results in the context of cellular death [46]. In addition, it has already been established that avidin doses of less than 100 μM have been shown not to affect chondrocyte viability or GAG loss in bovine cartilage explants [35].

A branched multi-arm avidin nanoconstruct was further evaluated *in vivo* in rat models, where doses below 300 μM were well-tolerated without detectable local or systemic toxicity. The construct demonstrated efficient joint residence and penetration into multiple intraarticular cell types, confirming its ability to distribute within cartilage and surrounding joint tissues. While no overt adverse effects were observed, a mild immunogenic response to the avidin backbone has been noted in prior studies and warrants consideration for translational applications [47].

Joint inflammation is driven by several factors, with nitric oxide and IL-6 being identified as two of the main ones. These, in turn, are major contributors to the cartilage destruction and progression of OA [48,49]. Av-bKGN (one dose of 1 μM) showed anti-inflammatory properties by reducing 25 to 50 % of IL-6 and NO^-^2 production in hCH and macrophages culture. Av-b5/bKGN nanocarrier exhibited a high cartilage uptake ratio of ~14X, which is significantly higher than its neutral counterpart (Ne-b5/bKGN, Ru = 3X) (Fig. 6A). The presence of SF impacted the intra-cartilage uptake by reducing it by almost a half (Fig. 6A). The SF constituent such as hyaluronic acid, albumin, and globulin can competitively bind with the avidin and hinder nanoparticle intra-cartilage transport [41]. A potential formation of an albumin-rich biocorona around avidin cannot be excluded as globular proteins behave similarly to nanoparticles. It has already been shown that biocoronas formation in SF significantly influences nanoparticles uptake into cartilage tissue and dictates their uptake into chondrocytes [40]. The reduction of Ne-b5/bKGN uptake in cartilage explant further suggested that the nature of interaction was not solely attributed to the charge interaction with the anionic SF constituents such as HA and albumin. In a previous study, it was also found that hydrophobic interactions were also substantial in the binding of cationic peptide prior to uptake in cartilage [41]. Still, even in SF, the uptake ratio in cartilage explant of Av-b5/bKGN (Ru = 8X) is 11.4X higher than the neutral avidin (Ru = 0.7X).

The long-term retention over 10 days of Av-b5/bKGN in cartilage explant highlighted the feature of charge-based systems for the delivery of a sustained therapeutic dose inside the anionic tissues. The neutral Ne-b5/bKGN and b5 both showed a low intra-cartilage retention after PBS desorption due to their lack of stabilizing cartilage interaction. This is corroborated by the transport chamber data where the effective diffusivities (Deff) in cartilage explant was an order of magnitude slower for the Av-based nanoconstruct compared to Ne (Fig. 6D).

Av-b5/bKGN demonstrated the properties of charge-based delivery system which could potentially lead into a drug depot-forming capability owing to its high uptake and long-term retention. In this study, it was found that a single dose of Av-bKGN significantly (p *<* 0.05) suppressed the GAG loss induced by IL-1α inflammation (Fig. 6E). The suppression level was significantly better than GAG loss in bKGN single dose treatment which resulted in a similar level as IL-1α only condition over 20 % GAGs loss (Fig. 6E), however, not reaching the level of noninflamed control. This could be attributed to the reduced administrated dose of KGN (10 μM) as well as the lack of additional pro-inflammatory mediator-Oncostatin M in the media. To better highlight the anti-catabolic KGN activity, the use of Oncostatin M synergistically with IL-1α is recommended [50,51]. Fig. 6E illustrates the benefit of using Av-bKGN as a cartilage-targeted delivery system, providing the same level of protection to the cartilage matrix as multiple doses of free bKGN with no significative difference. However, the limited concentration incorporated into the delivery system of 10 μM is a drawback of the current approach. The multi-arm avidin nano-construct developed by He et *al.* [15] enables the drug loading content of KGN to be increased to 100 μM maintaining a safe concentration of Av (*<*1 μM), and producing promising results. High uptake of polycationic carriers in cartilage explant can affect its swelling and mechanical properties altering chondrocyte metabolism and health [52]. However, our work and previous works using Av as carriers showed that it has no adverse effects on tissue or cell health (Fig. 5C and S12) or electro-mechanical properties at the therapeutical dosage [52,53].

Although the reported bKGN payload per construct (~10 μM) is lower than that achievable with multi-arm avidin (~100 μM), this level of loading appears sufficient for *in vitro* and *ex vivo* studies, where effective modulation can be achieved at micromolar concentrations. In these controlled settings, the delivered dose falls within ranges commonly reported to elicit biological responses for peptide- and small-molecule therapeutics [54]. Importantly, sustained joint residence and cartilage uptake/retention, rather than maximal instantaneous payload, are primary determinants of IA efficacy [55]. For *in vivo* IA applications—where joint volume, clearance kinetics, and tissue diffusion impose additional constraints—higher payload loadings on the order of ~100 μM will therefore be considered to ensure adequate local exposure and therapeutic efficacy. Further *in vivo* studies will investigate the effects of a nominal concentration of Av-bKGN (100 μM) in a preclinical OA animal model. Consistent with this rationale, multi-arm avidin systems carrying a nominal KGN loading of ~100 μM have been shown, following a single administration, to produce enhanced biological activity *ex vivo*, including suppression of cytokine-induced catabolic pathways associated with OA [22]. Furthermore, incorporating multi-arm PEG into the nanoconstruct is beneficial, as it reduces immunogenic opsonisation and/or biocorona formation, thereby supporting prolonged joint residence, functional efficacy and *in vivo* safety [47].

As a perspective for evaluating the immunogenicity of avidin-based or cartilage-targeted nanocarriers, three-dimensional organoid models represent a promising approach, offering several key advantages over conventional *in vitro* systems. First, organoids derived from human pluripotent stem cells or patient-specific primary cells enhance human relevance and enable more accurate prediction of clinically relevant immune responses [56]. Second, organoids preserve native three-dimensional tissue architecture, cellular polarity, and extracellular matrix interactions, all of which are critical determinants of immune recognition, antigen presentation, and inflammatory signalling [56]. Third, when integrated with immune cell co-culture strategies, organoid platforms enable tissue-specific insights into innate and adaptive immune activation, including cytokine release and immune cell recruitment [57]. Finally, organoid-based immunogenicity assessment aligns with the principles of the 3Rs (Replacement, Reduction, and Refinement), reducing dependence on animal models while maintaining a high degree of physiological and immunological complexity [58].

To resume, our projec’s novelty lay in the innovative synthesis of KGN from its precursors via biotinylation, as well as in the metabolization study that led us to discover a new KGN degradation product (ISO) in a simulated arthritic environment for the first time. Similarly, in the study by Vedadghavami et *al.* [41], where cationic peptide uptake in cartilage explant was reduced in the presence of SF due to the predominant hydrophobic interactions—principally due to competitive binding-with albumin and globulins—our study also found that Av-nanocarrier uptake was decreased. Nevertheless, Av-based nanocarriers demonstrate greater cartilage uptake and retention in SF than their neutral counterparts (Ne), demonstrating the potential of electrostatic interactions between delivery systems and the cartilage matrix for targeted delivery approaches. The *ex vivo* results of IL-1α metabolism suppression in bovine cartilage explants are consistent with the precedents *in vitro* suppression of IL-6 production on hCHs proving the potential anti-inflammatory/anti-catabolic properties of our Av-bKGN for OA treatment. The combination of a protein-based delivery system (Av) with DMOAD modulating cartilage metabolism (bKGN) seems to be a promising approach for intra-articular treatment of OA. Further investigation is required in preclinical OA animal model to demonstrate its preclinical safety (dose-response study) and efficacy.

## 5. Conclusion

This study demonstrates the synthesis of kartogenin through bio-tinylation, followed by its conjugation to cationic avidin to create a tetrameric, protein-based conjugate, Av-bKGN. The avidin-based system exhibits anti-inflammatory properties and is not toxic on human chondrocytes. It exhibits a higher intra-cartilage uptake and retention, both in physiological and simulated synovial fluid conditions, compared to its neutral counterpart. The diffusivity of this cationic globular protein is dictated by its small size (*<*10 nm) and its positive net charge (+20), allowing it to interact electrostatically with the cartilage matrix. A single dose of Av-bKGN (10 μM) protects the cartilage matrix from cytokine-induced degradation in the same way as several doses of the free drug, although not reaching the GAG loss level of the untreated control group, likely due to the limited drug loading capacity of the system. Notably, our study further identified a novel kartogenin degradation product for the first time, with NO inhibitory activity *in vitro*. In the future, *in vitro/ex vivo* studies are warranted to investigate if ISO would replicate KGN−s well-known disease-modifying osteoarthritic properties. Similarly, the bioavailability and pharmacokinetic profiles of ISO would require further investigation.

## Supplementary Material

1

## Data Availability

Data will be made available on request.

## Appendix A. Supplementary data

Supplementary data to this article can be found online at https://doi.org/10.1016/j.jddst.2026.108162.
