# Supplementary material for: Protein-based nanocarrier delivering kartogenin derivative to cartilage matrix for intra-articular treatment of osteoarthritis
Source: J Drug Deliv Sci Technol. Author manuscript; Available in PMC 2026 Jun 16. (PMC13267916; doi:10.1016/j.jddst.2026.108162)
Supplement: 1 [file NIHMS2178816-supplement-1.docx]

**SUPPLEMENTARY INFORMATION**

Protein-based nanocarrier delivering kartogenin derivative to cartilage matrix for intra-articular treatment of osteoarthritis

^1,2^[Luca Morici](https://pubmed.ncbi.nlm.nih.gov/?term=Morici+L&cauthor_id=38163526), ^1,2^Sebastien Jenni, ^3^Bill Hakim, ^1,2^Carlos Rodríguez-Nogales, ^1,2^Eric Allémann, ^3^Ambika G. Bajpayee, ^1,2^[Olivier Jordan](https://pubmed.ncbi.nlm.nih.gov/?term=Jordan+O&cauthor_id=38163526)*

1 School of Pharmaceutical Sciences, University of Geneva, Rue Michel-Servet 1, 1211 Geneva 4, Switzerland

2 Institute of Pharmaceutical Sciences of Western Switzerland, Rue Michel-Servet 1, 1211 Geneva 4, Switzerland

3 Department of Bioengineering, Northeastern University, 805 Columbus Avenue, Boston, MA 02120, USA

* Corresponding authors

Olivier.Jordan@unige.ch

INDEX

1. ^1^H-NMR of intermediate product 1
2. ^13^C-NMR of intermediate product 1
3. HRMS ESI positive of intermediate product 1
4. HRMS ESI negative of intermediate product 1
5. ^1^H-NMR of biotin-PEG_2_-kartogenin (b-KGN)
6. ^13^C-NMR of biotin-PEG_2_-kartogenin (b-KGN)
7. HRMS ESI positive of biotin-PEG_2_-kartogenin (b-KGN)
8. UHPLC peak representative chromatogram at 280 nm of b-KGN after 6 h of incubation at 37°C in PBS
9. Calibration curve of KGN
10. Synthesis and calibration curve of ISO
11. UHPLC peak representative chromatogram at 280 nm of 4-aminobiphenyl
12. LDH cytotoxicity assay
13. Calibration curve of nitrite
14. Calibration curve of IL-6
15. Calibration curve of b5
16. Calibration curve of Av-b5
17. Calibration curve of Ne-b5
18. DMMB calibration curves for supernatant and digested explant
19. AF4 fractograms of the avidin-albumin interaction
20. ^1^H-NMR of intermediate product 1

**Figure S1.** ^1^H NMR (600 MHz) of intermediate product 1 in DMSO-*d6*.

1. ^1^C-NMR of intermediate product 1

**Figure S2.** ^13^C NMR (151 MHz) of intermediate product 1 in DMSO-d6.

1. HRMS ESI positive of intermediate product 1


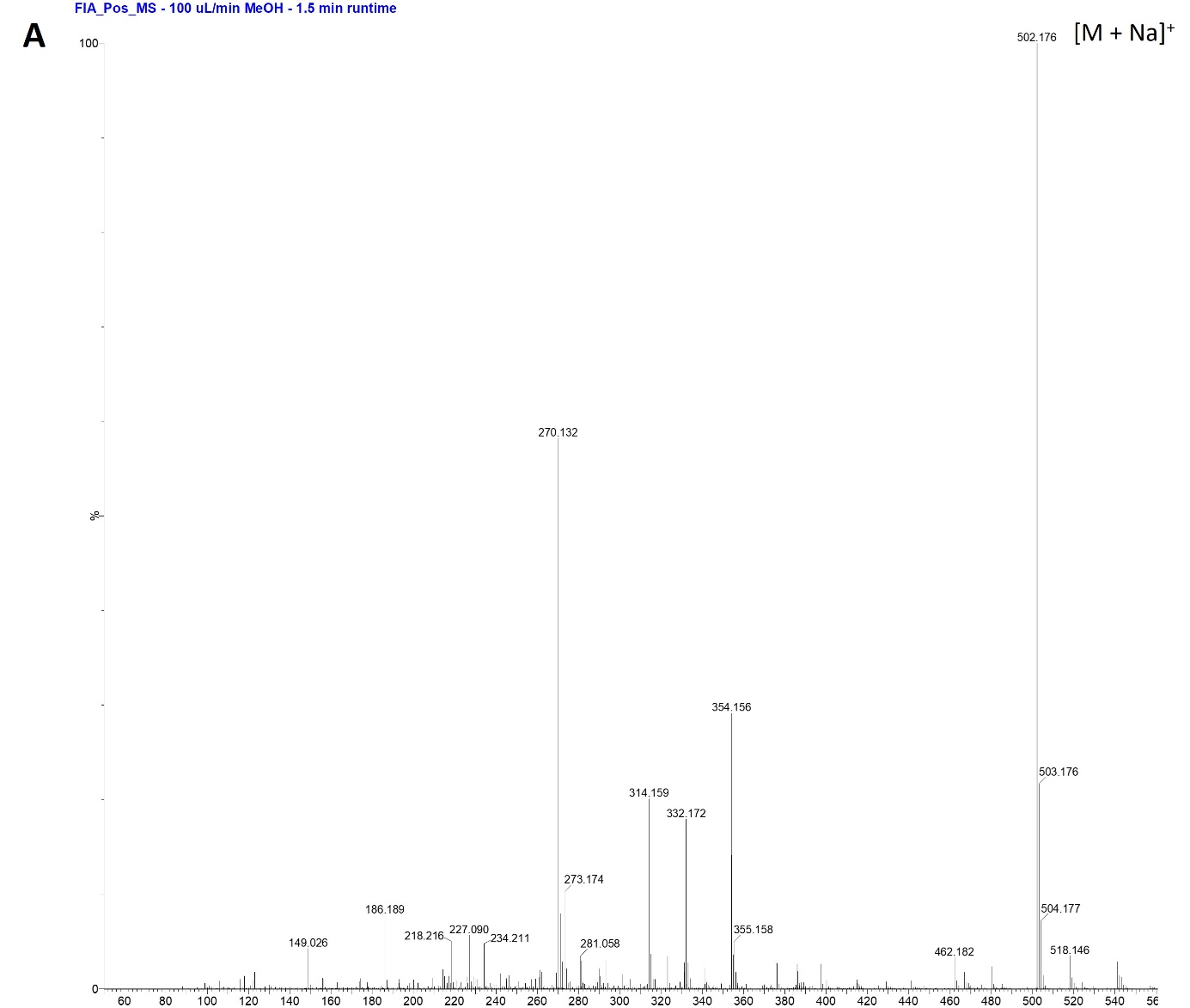


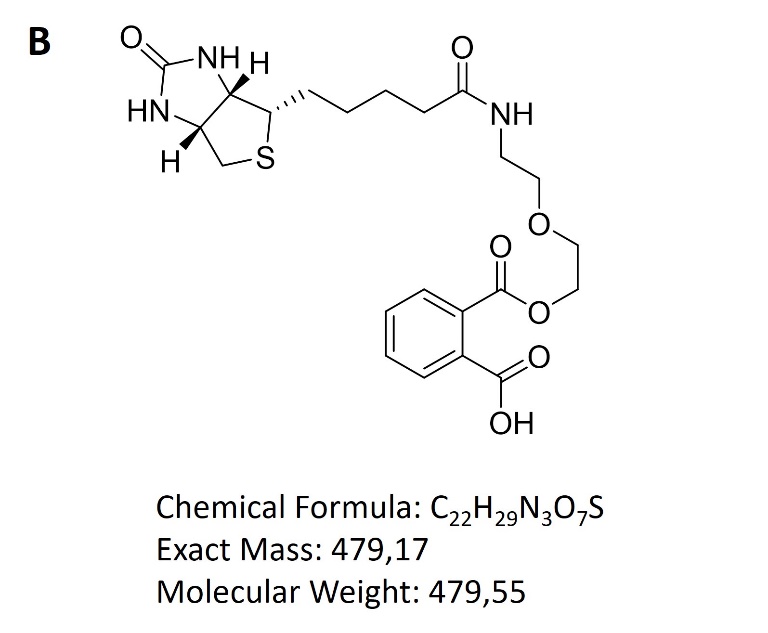


**Figure S3.** HRMS ESI positive of the intermediate product 1 (A) and its molecular structure and chemical formula (B).

1. HRMS ESI negative of intermediate product 1


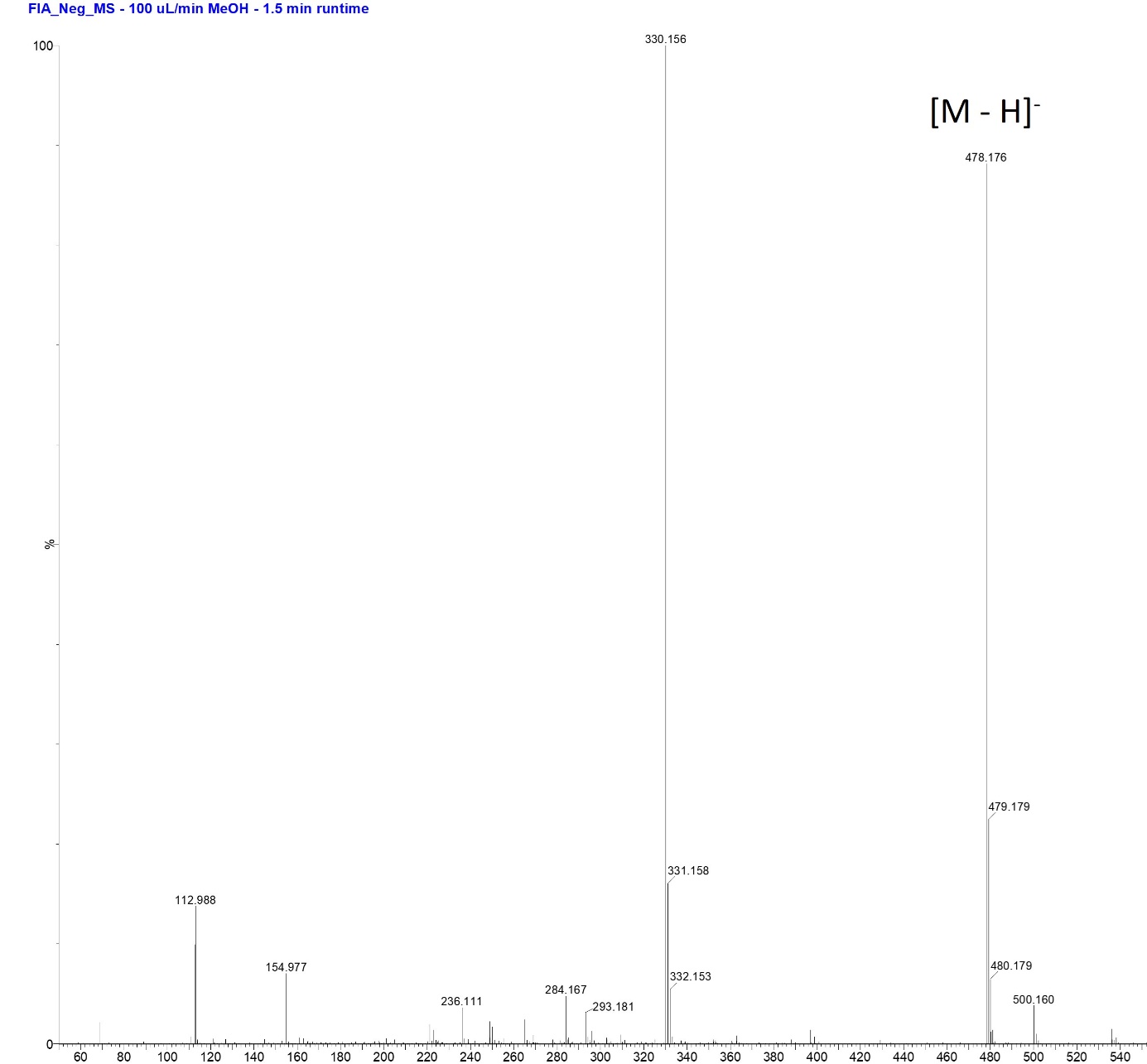


**Figure S4.** HRMS ESI negative of the intermediate product 1.

1. ^1^H-NMR of biotin-PEG2-kartogenin (b-KGN)

**Figure S5.** ^1^H NMR (600 MHz) of biotin-PEG2-kartogenin in DMSO-*d6*.

1. ^1^C-NMR of biotin-PEG2-kartogenin (b-KGN)

**Figure S6.** ^13^C NMR (151 MHz) of biotin-PEG2-kartogenin in DMSO-d6.

1. HRMS ESI positive of biotin-PEG_2_-kartogenin (b-KGN)


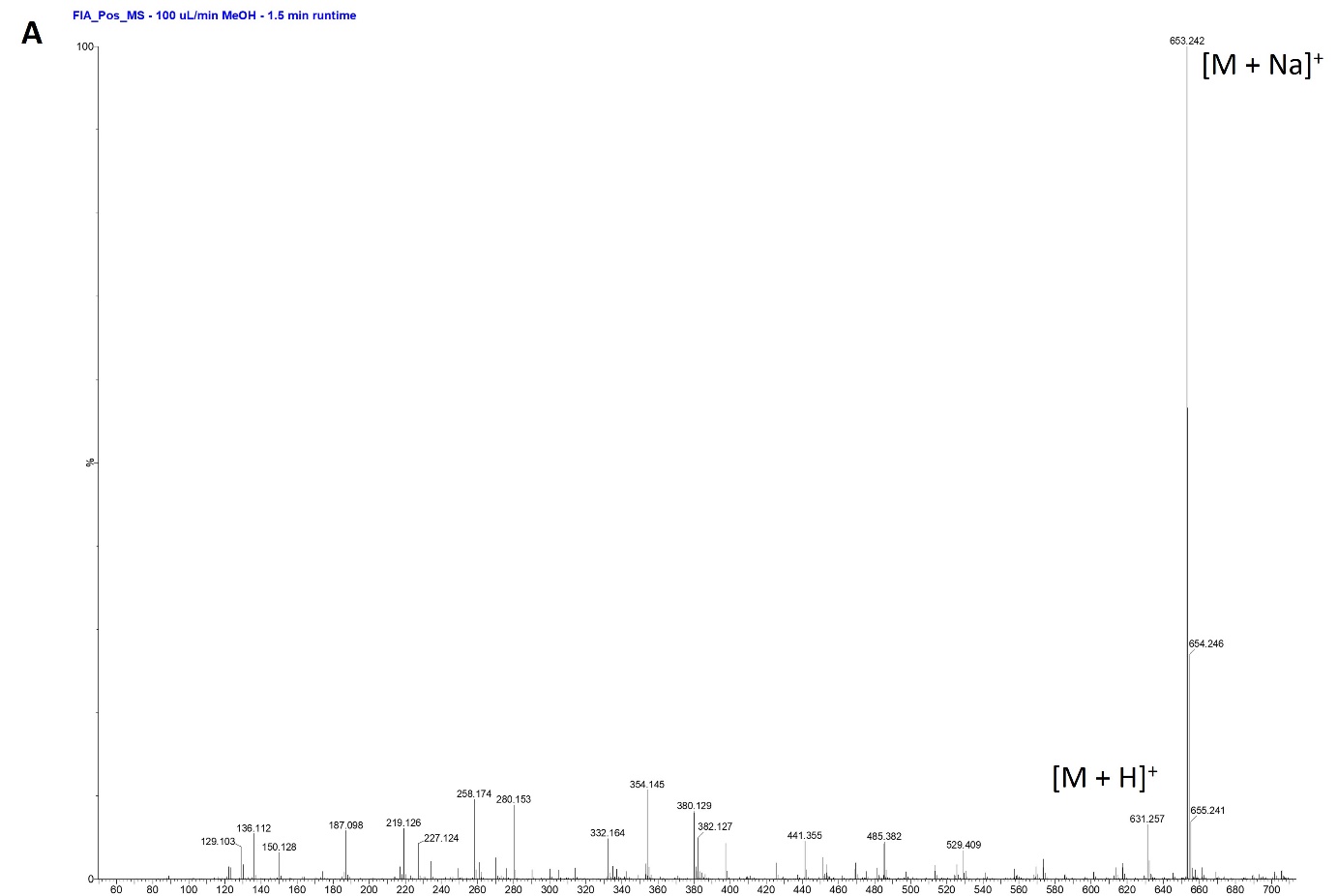


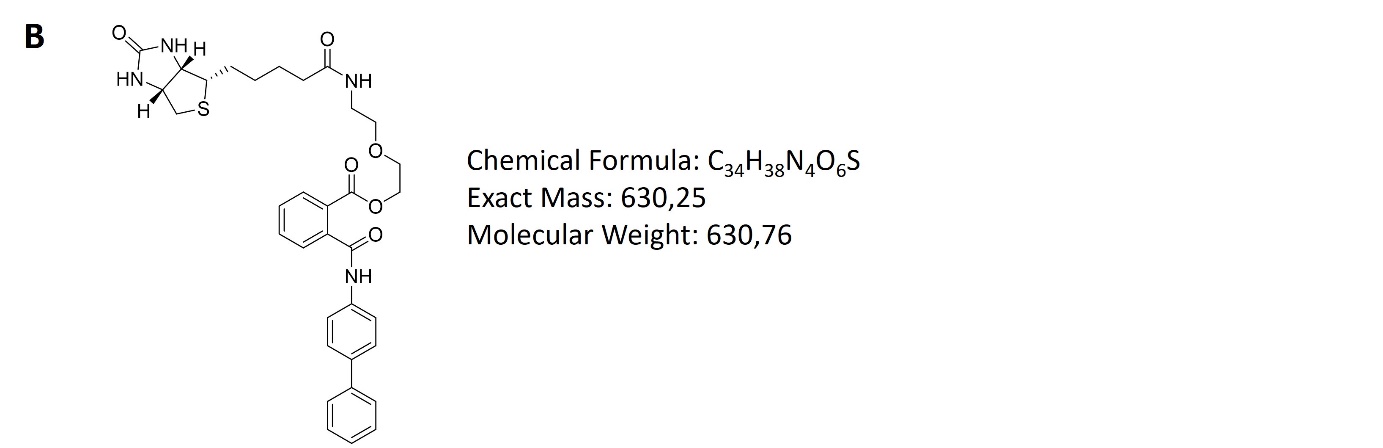


**Figure S7.** HRMS ESI positive of the final product (biotin-PEG_2_-kartogenin or b-KGN)(A) and its molecular structure and chemical formula (B).

1. UHPLC peak representative chromatogram at 280 nm of b-KGN after 24 h of incubation at 37°C in PBS.

*
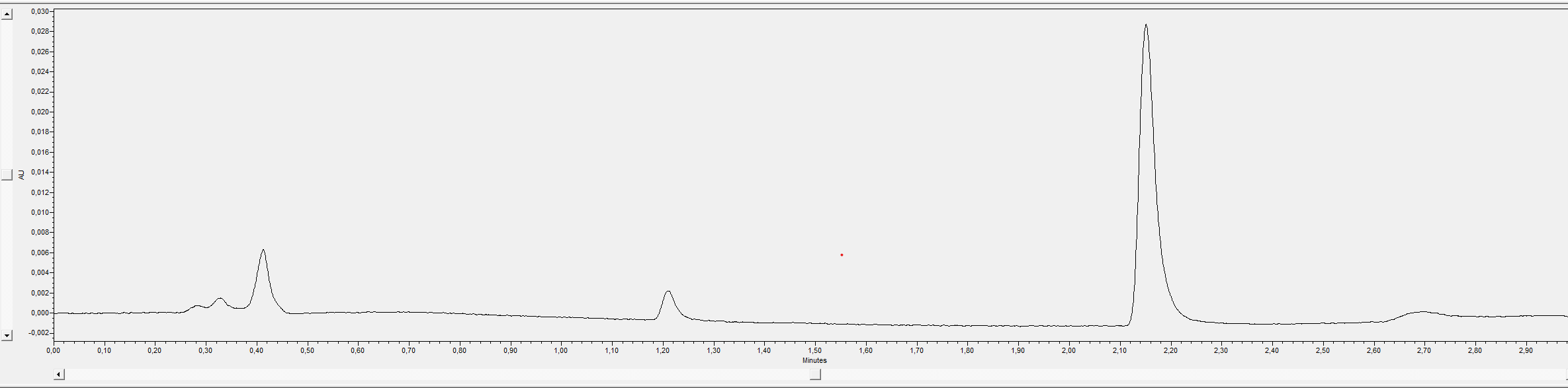
*

**C**

*
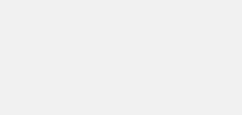
*

**B**

**A**

**Figure S8.** UHPLC peak representative chromatogram at 280 nm of b-KGN after 24 h of incubation at 37°C in PBS. b-KGN (A) eluted at 0.413 min, KGN (B) at 1.225 min and ISO (C) at 2.146 min.

1. Calibration curve of KGN


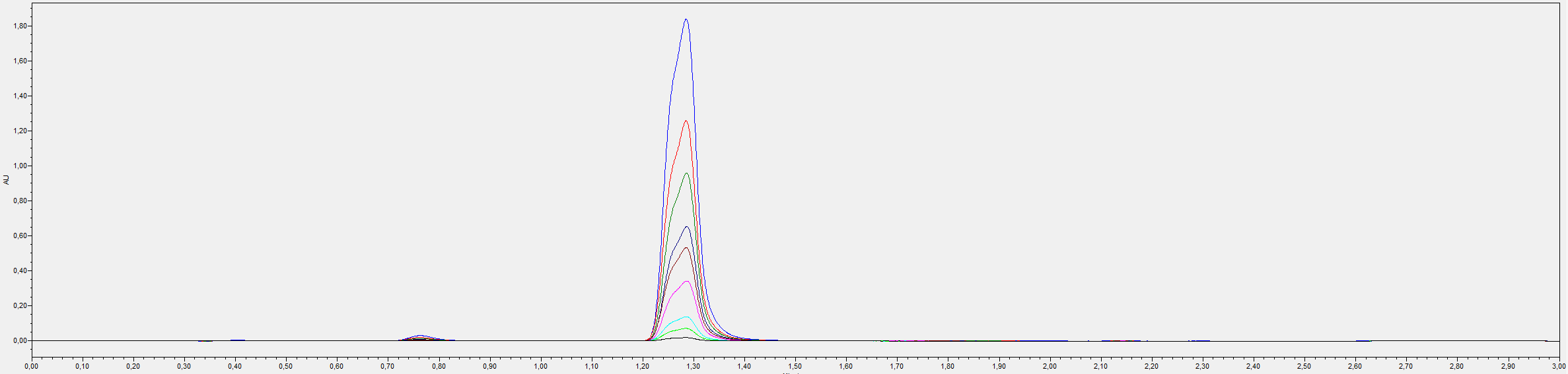

**Figure S9.** Representative calibration curve of KGN at 280 nm. UHPLC peak of ISO eluted at 1.225 min.

1. Synthesis and calibration curve of ISO

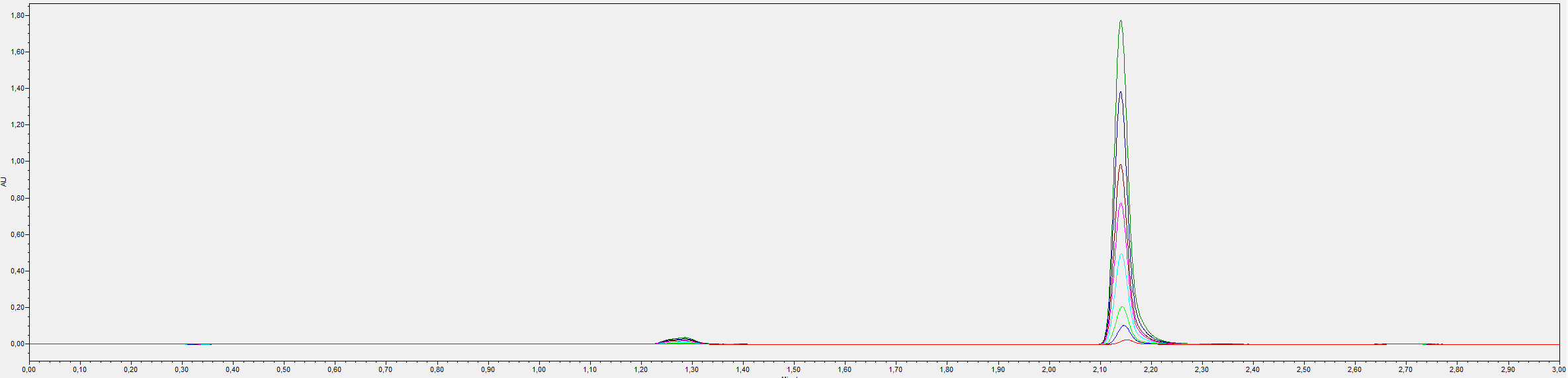

**Figure S10**. Representative calibration curve of ISO at 280 nm. UHPLC peak of ISO eluted at 2.146 min. Our previous work already reported the characterisation of this ISO derivative compound using ^1^H-NMR, ^13^C-NMR and ESI-MS [10].

1. UHPLC peak representative chromatogram at 280 nm of 4-aminobiphenyl

**Figure S11**. UHPLC peak of 4-aminobiphenyl 280 nm (tR: 0.782 min).

1. LDH cytotoxicity assay


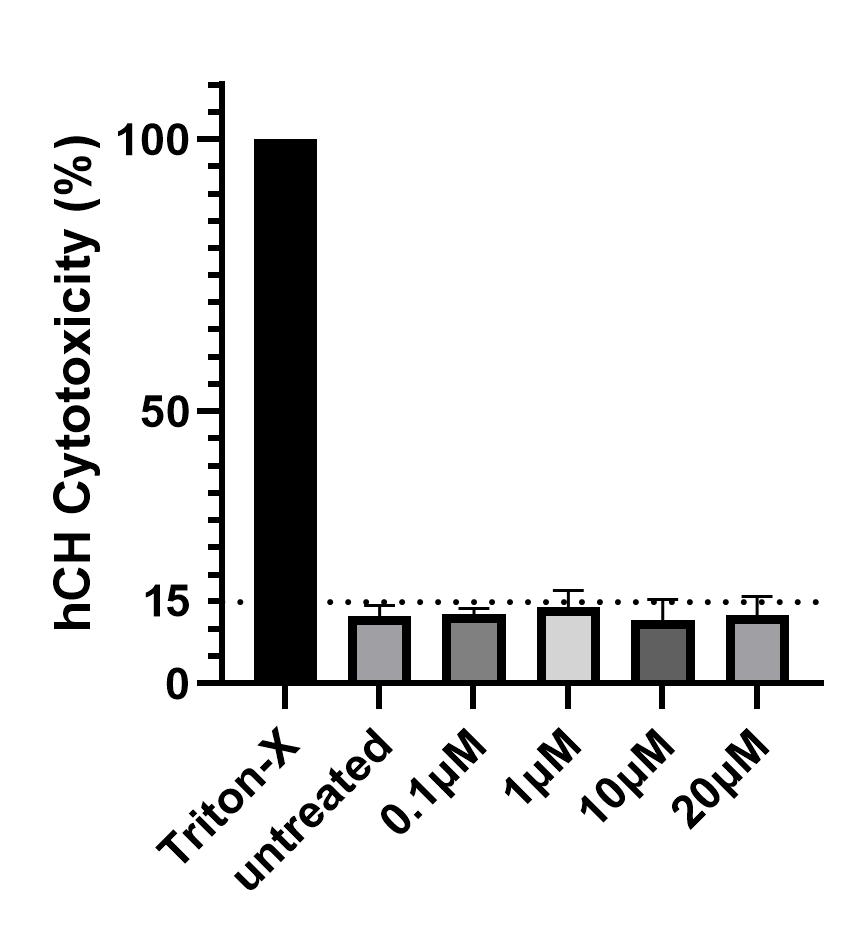


**Figure S12**. LDH assay on hCH treated with Av-bKGN at concentrations ranging from 0.1 to 20 μM.

1. Calibration curve of nitrite

*
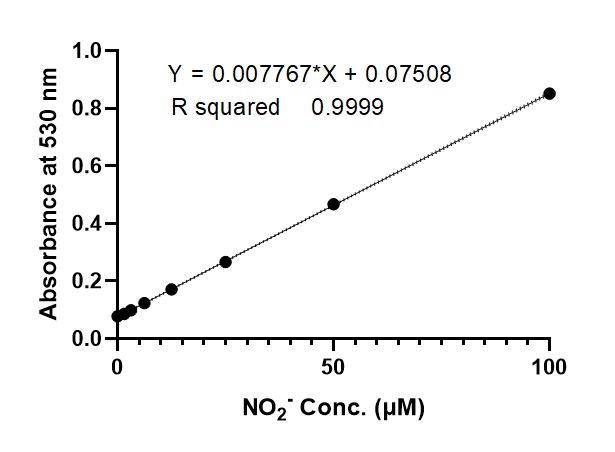
*

| NO_2_^-^(μM) | Absorbance at 530 nm |
| --- | --- |
| 100 | 0,851 |
| 50 | 0,467 |
| 25 | 0,266 |
| 12,5 | 0,171 |
| 6,25 | 0,124 |
| 3,13 | 0,099 |
| 1,56 | 0,086 |
| 0 | 0,078 |

**Figure S13**. Calibration curve of nitrites (NO_2_^-^) from 0 to 100 μM.

1. Calibration curve of IL-6


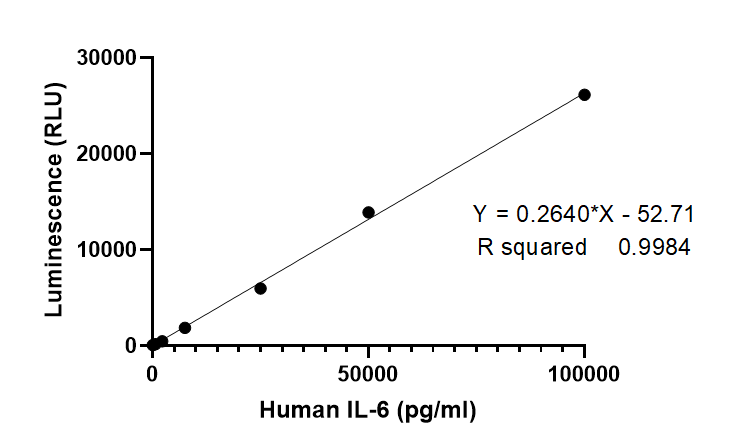


| Human IL-6 (pg/ml) | Luminescence (RLU) |
| --- | --- |
| 100000 | 26134 |
| 50000 | 13887 |
| 25000 | 5956 |
| 7500 | 1867 |
| 2250 | 470 |
| 675 | 163 |
| 103 | 64 |
| 60,8 | 37 |

**Figure S14**. Calibration curve of human IL-6 from 60.8 to 100000 pg/mL performed by Lumit™ IL-6 Immunoassay.

1. Calibration curve of b5

| b5 (mg/mL) | Fluorescence Intensity |
| --- | --- |
| 0,1 | 9686 |
| 0,05 | 4131 |
| 0,025 | 2239 |
| 0,0125 | 1018 |
| 0,0063 | 544 |
| 0,00315 | 292 |

**Figure S15**. Calibration curve of b5 from 0.00315 to 0.1mg/mL.

1. Calibration curve of Av-b5/bKGN

| Av-b5/bKGN (mg/mL) | Fluorescence Intensity |
| --- | --- |
| 0,1 | 93598 |
| 0,05 | 45402 |
| 0,025 | 21509 |
| 0,0125 | 9119 |
| 0,0063 | 4103 |
| 0,00315 | 1967 |

**Figure S16**. Calibration curve of Av-b5/bKGN from 0.00315 to 0.1mg/mL.

1. Calibration curve of Ne-b5/bKGN

| Ne-b5/bKGN (mg/mL) | Fluorescence Intensity |
| --- | --- |
| 0,1 | 7799 |
| 0,025 | 1065 |
| 0,0125 | 437 |
| 0,0063 | 334 |
| 0,00315 | 43 |

**Figure S17**. Calibration curve of Ne-b5/bKGN from 0.00315 to 0.1mg/mL.

1. DMMB calibration curves for supernatant and digested explant

| Chondroitin sulfate in DMEM (μg/mL) | Absorbance at 520 nm |
| --- | --- |
| 25 | 0,271 |
| 12,5 | 0,218 |
| 6,25 | 0,188 |
| 3,125 | 0,178 |
| 1,5626 | 0,169 |
| 0 | 0,158 |

| Chondroitin sulfate in TRIS buffer (μg/mL) | Absorbance at 520 nm |
| --- | --- |
| 100 | 0,35 |
| 50 | 0,278 |
| 25 | 0,233 |
| 12,5 | 0,202 |
| 6,25 | 0,195 |
| 3,125 | 0,188 |
| 1,5626 | 0,183 |
| 0 | 0,179 |

**Figure S18.** Calibration curves realised in DMEM (A) for the quantification of GAGs in supernatants and in TRIS buffer (B) for the quantification of GAGs in digested explants.

1. **AF4 fractograms of the avidin-albumin interaction

|  | Avidin | Albumin | Avidin + Albumin |
| --- | --- | --- | --- |
| Elution time (min) | 4.3 | 5.1 | 6.5 |
| Radius of gyration (Rg) (nm) | 6.9 | 9.3 | 14.6 |

**Figure S19.** AF4 fractograms of avidin, albumin and avidin-albumin interaction.
